# Mutated and Bacteriophage T4 Nanoparticle Arrayed F1-V Immunogens from *Yersinia pestis* as Next Generation Plague Vaccines

**DOI:** 10.1371/journal.ppat.1003495

**Published:** 2013-07-11

**Authors:** Pan Tao, Marthandan Mahalingam, Michelle L. Kirtley, Christina J. van Lier, Jian Sha, Linsey A. Yeager, Ashok K. Chopra, Venigalla B. Rao

**Affiliations:** 1 Department of Biology, The Catholic University of America, Washington, District of Columbia, United States of America; 2 Department of Microbiology and Immunology, The University of Texas Medical Branch, Galveston, Texas, United States of America; 3 Institute of Human Infections and Immunity, The University of Texas Medical Branch, Galveston, Texas, United States of America; 4 Galveston National Laboratory, The University of Texas Medical Branch, Galveston, Texas, United States of America; 5 Sealy Center for Vaccine Development, The University of Texas Medical Branch, Galveston, Texas, United States of America; Osaka University, Japan

## Abstract

Pneumonic plague is a highly virulent infectious disease with 100% mortality rate, and its causative organism *Yersinia pestis* poses a serious threat for deliberate use as a bioterror agent. Currently, there is no FDA approved vaccine against plague. The polymeric bacterial capsular protein F1, a key component of the currently tested bivalent subunit vaccine consisting, in addition, of low calcium response V antigen, has high propensity to aggregate, thus affecting its purification and vaccine efficacy. We used two basic approaches, structure-based immunogen design and phage T4 nanoparticle delivery, to construct new plague vaccines that provided complete protection against pneumonic plague. The NH_2_-terminal β-strand of F1 was transplanted to the COOH-terminus and the sequence flanking the β-strand was duplicated to eliminate polymerization but to retain the T cell epitopes. The mutated F1 was fused to the V antigen, a key virulence factor that forms the tip of the type three secretion system (T3SS). The F1mut-V protein showed a dramatic switch in solubility, producing a completely soluble monomer. The F1mut-V was then arrayed on phage T4 nanoparticle via the small outer capsid protein, Soc. The F1mut-V monomer was robustly immunogenic and the T4-decorated F1mut-V without any adjuvant induced balanced T_H_1 and T_H_2 responses in mice. Inclusion of an oligomerization-deficient YscF, another component of the T3SS, showed a slight enhancement in the potency of F1-V vaccine, while deletion of the putative immunomodulatory sequence of the V antigen did not improve the vaccine efficacy. Both the soluble (purified F1mut-V mixed with alhydrogel) and T4 decorated F1mut-V (no adjuvant) provided 100% protection to mice and rats against pneumonic plague evoked by high doses of *Y. pestis* CO92. These novel platforms might lead to efficacious and easily manufacturable next generation plague vaccines.

## Author Summary

Plague caused by *Yersinia pestis* is a deadly disease that wiped out one-third of Europe's population in the 14th century. The organism is listed by the CDC as Tier-1 biothreat agent, and currently, there is no FDA-approved vaccine against this pathogen. Stockpiling of an efficacious plague vaccine that could protect people against a potential bioterror attack has been a national priority. The current vaccines based on the capsular antigen (F1) and the low calcium response V antigen, are promising against both bubonic and pneumonic plague. However, the polymeric nature of F1 with its propensity to aggregate affects vaccine efficacy and generates varied immune responses in humans. We have addressed a series of concerns and generated mutants of F1 and V, which are completely soluble and produced in high yields. We then engineered the vaccine into a novel delivery platform using the bacteriophage T4 nanoparticle. The nanoparticle vaccines induced robust immunogenicity and provided 100% protection to mice and rats against pneumonic plague. These highly efficacious new generation plague vaccines are easily manufactured, and the potent T4 platform which can simultaneously incorporate antigens from other biothreat or emerging infectious agents provides a convenient way for mass vaccination of humans against multiple pathogens.

## Introduction

Plague, also known as Black Death, is one of the deadliest infectious diseases known to mankind. *Yersinia pestis*, the etiologic agent of plague, is a Gram-negative bacterium transmitted from rodents to humans via fleas [1]. The bite of an infected flea results in bubonic plague which can then develop into secondary pneumonic plague, resulting in person-to-person transmission of the pathogen through infectious respiratory droplets [2]. Pneumonic plague can also be caused by direct inhalation of the aerosolized *Y. pestis*, leading to near 100% death of infected individuals within 3–6 days [2], [3]. Due to its exceptional virulence and relative ease of cultivation, aerosolized *Y. pestis* poses one of the greatest threats for deliberate use as a biological weapon [4]. Since the disease spreads rapidly, the window of time available for post-exposure therapeutics is very limited, usually 20–24 h after the appearance of symptoms [3]. Although levofloxacin has recently been approved by the Food and Drug Administration (FDA) for all forms of plague (http://www.fda.gov/NewsEvents/Newsroom/PressAnnouncements/ucm302220.htm), prophylactic vaccination is one of the most effective means to reduce the risk of plague.

Stockpiling of an efficacious plague vaccine has been a national priority since the 2001 anthrax attacks but no vaccine has yet been licensed. Previously, a killed whole cell (KWC) vaccine was in use in the United States, and a live attenuated plague vaccine (EV76) is still in use in the states of former Soviet Union [5]. However, the need for multiple immunizations, high reactogenicity, and insufficient protection made the KWC vaccine undesirable for mass vaccination, and, consequently, it was discontinued in the United States [6]. In fact, the live-attenuated vaccine may not meet FDA approval because of the highly infectious nature of the plague bacterium and the virulence mechanisms of vaccine strains have not been fully understood [6], [7]. A cautionary tale related to this is the recent fatality of a researcher as a result of exposure to the attenuated pigmentation-minus *Y. pestis* strain, KIM/D27 (http://en.wikipedia.org/wiki/Malcolm_Casadaban].

**Figure 1:**
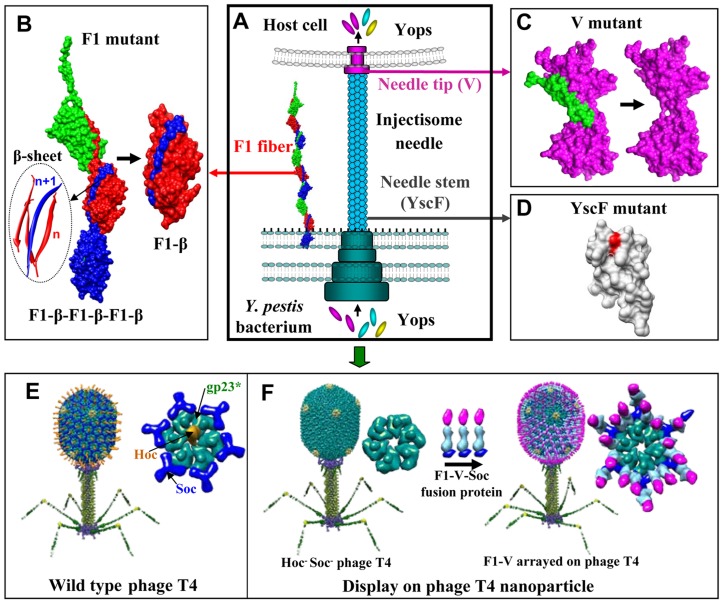
New plague immunogen designs. Schematic of various approaches used to design plague immunogens. See text for details. (**A**) *Y. pestis* surface components targeted for vaccine design. F1 is the structural unit of the capsular layer. V forms a pore at the tip of the injectisome needle and facilitates translocation of Yops into the host cell. YscF is the structural unit of the injectisome needle. (**B**) Reorientation of the NH_2_-terminal β-strand of F1 to generate monomeric F1. “n” and “n+1” refer to the F1 subunits the β-strands belong to; the red strands to “n” subunit and the blue strand to the “n+1” subunit. (**C**) Deletion of the putative immunomodulatory sequence (aa residues 271–300) of V antigen. (**D**) Mutagenesis of Asn35 and Ile67 to produce an oligomerization deficient YscF. (**E**) Structural model of bacteriophage T4. The enlarged capsomer shows the major capsid protein gp23* (green; “*” represents the cleaved form) (930 copies), Soc (blue; 870 copies), and Hoc (yellow; 155 copies). Yellow subunits at the five-fold vertices correspond to gp24*. The portal vertex (not visible in the picture) connects the head to the tail. (**F**) Display of F1mut-V-Soc fusion protein on the Hoc^−^ Soc^−^ phage particle. Models of the enlarged capsomers before and after F1mut-V display are shown.

The focus in the past two decades, thus, has shifted to the development of recombinant subunit vaccines [3], [6], [8], [9] containing two *Y. pestis* virulence factors, the capsular protein (Caf1 or F1; 15.6 kDa, Figure 1A and B) and the low calcium response V antigen (LcrV or V; 37.2 kDa, Figure 1A and C), which is a component of the type 3 secretion system (T3SS). F1 assembles into flexible linear fibers via a chaperone/*usher* mechanism [10], forming a capsular layer that allows *Y. pestis* to adhere to the host cell and escape phagocytosis [11] (Figure 1A and 1B). The V antigen forms a “pore” at the tip of the “injectisome” structure of the T3SS needle, creating a channel that delivers a range of virulence factors, also known as the *Yersinia* outer membrane proteins (Yops), into the host cytosol (Figure 1A) [12]. The V antigen is also critical for impairment of host's phagocytic responses [13]. Abrogation of these functions by F1 and V antibodies appears to be one of the mechanisms leading to protection of the host against lethal *Y. pestis* infection.

Two types of F1/V recombinant vaccines have been under investigation, one containing a mixture of F1 and V antigens [14], and another, a single F1-V fusion protein [15], [16]. Although both induce protective immunity against *Y. pestis* challenge in rodent and cynomolgus macaque models, protection of African Green monkeys was insufficient and highly variable [6], [17]. A phase I clinical trial in humans showed that a vaccine consisting of a mixture of F1 and V proteins was immunogenic, however, the antibody titers varied over a wide range leading to concerns about the consistency of vaccine efficacy [18].

One of the problems associated with the current plague vaccines is that the naturally fibrous F1 polymerizes into heterodisperse aggregates, compromising the quality and overall efficacy of the vaccines [15], [19], [20], [21], [22]. Second, the subunit vaccines do not induce adequate cell-mediated immune responses that also appear to be essential for optimal protection against plague [23]. Third, it is unclear if inclusion of other *Y. pestis* antigens such as the YscF, the structural unit of the injectisome needle (Figure 1A and 1D), can boost the potency of the F1/V vaccines. This is particularly important as F1-minus strains of *Y. pestis* exist in nature which are as virulent as the wild-type strains [24], [25] and significant diversity in the LcrV sequence of these strains might render the current F1/V vaccines ineffective [26], [27]. Finally, the reported immunosuppressive property of V antigen [13], [28] and whether it could compromise the innate immunity of humans, is a significant concern. These questions must be addressed to generate a next generation plague vaccine that could pass licensing requirements, as well as be manufactured relatively easily for stockpiling.

Recently, we have developed a novel vaccine delivery system using the bacteriophage T4 nanoparticle [29], [30], [31], [32]. The T4 capsid (head) is an elongated icosahedron, 120 nm long and 86 nm wide, composed of three essential capsid proteins: a major capsid protein, gp23*; vertex protein, gp24*; and a portal protein, gp20 (Figure 1E). It is decorated with two non-essential proteins, Soc, the small outer capsid protein, and Hoc, the highly antigenic outer capsid protein. Binding sites for these proteins appear following head “expansion,” a major conformational change that increases the outer dimensions of the capsid by ∼15% and inner volume by ∼50% [33].

Approximately 870 molecules of the tadpole- shaped Soc protein (9 kDa) assemble into trimers at the quasi three-fold axes, clamping to adjacent capsomers and forming a reinforced cage around the shell (Figure 1E) [34]. This stabilizes an already stable head that can withstand harsh extracellular environment (e.g., pH 11) [34]. Hoc, on the other hand, is a linear “fiber” containing a string of four domains, three of which are immunoglobulin (Ig)-like [35]. One hundred and fifty five copies of Hoc fibers, with their NH_2_-termini projected at ∼160 Å distance from the capsid assemble at the center of each capsomer (Figure 1E). Hoc binds to bacterial surfaces, apparently enriching the phage near its host for infection [36]. Although Soc and Hoc provide survival advantages, they are completely dispensable under laboratory conditions showing no significant effect on phage productivity or infectivity [37]. Purified Soc (or Hoc) protein binds to Hoc^−^ Soc^−^ capsid with high specificity and nanomolar affinity, properties that are not compromised by attachment of a pathogen antigen at the NH_2_- and COOH-termini [29], [30], [31], [32]. Individual domains, or full-length proteins as large as 90 kDa, or multilayered oligomeric complexes of >500 kDa fused to Soc can be arrayed on T4 capsid, making it a robust antigen delivery platform [29], [30].

Here, we describe two basic approaches to generate next generation plague vaccines, structure-based immunogen design and T4 nanoparticle delivery (Figure 1). We designed an F1 mutant that retained the T cell epitopes but folded into a soluble monomer rather than into an insoluble fiber (Figure 1B). The mutated F1 was fused to V antigen to produce a bivalent F1mut-V immunogen that was also expressed as a soluble monomer. We then constructed an oligomerization deficient YscF mutant (Figure 1D) as well as a V mutant without the putative immunomodulatory sequence (Figure 1C). The mutated antigens were fused to Soc and arrayed on phage T4 nanoparticle (Figure 1F). The F1mut-V monomer induced robust immunogenicity, and the T4-decorated F1mut-V without any adjuvant, in addition, induced balanced T_H_1 and T_H_2 responses. Both the soluble and T4 decorated F1mut-V provided 100% protection to mice and rats against intranasal challenge with high doses of *Y. pestis* CO92. Inclusion of YscF showed a slight enhancement in the potency of F1-V plague vaccine, whereas replacement of V with V10 mutant, which lacks the putative immunosuppressive sequence, did not significantly alter vaccine efficacy. These results provided new insights into plague vaccine design and produced next generation plague vaccine candidates by overcoming some of the concerns associated with the current subunit vaccines.

## Results

### Designing a soluble monomeric F1 mutant

**Figure 2:**
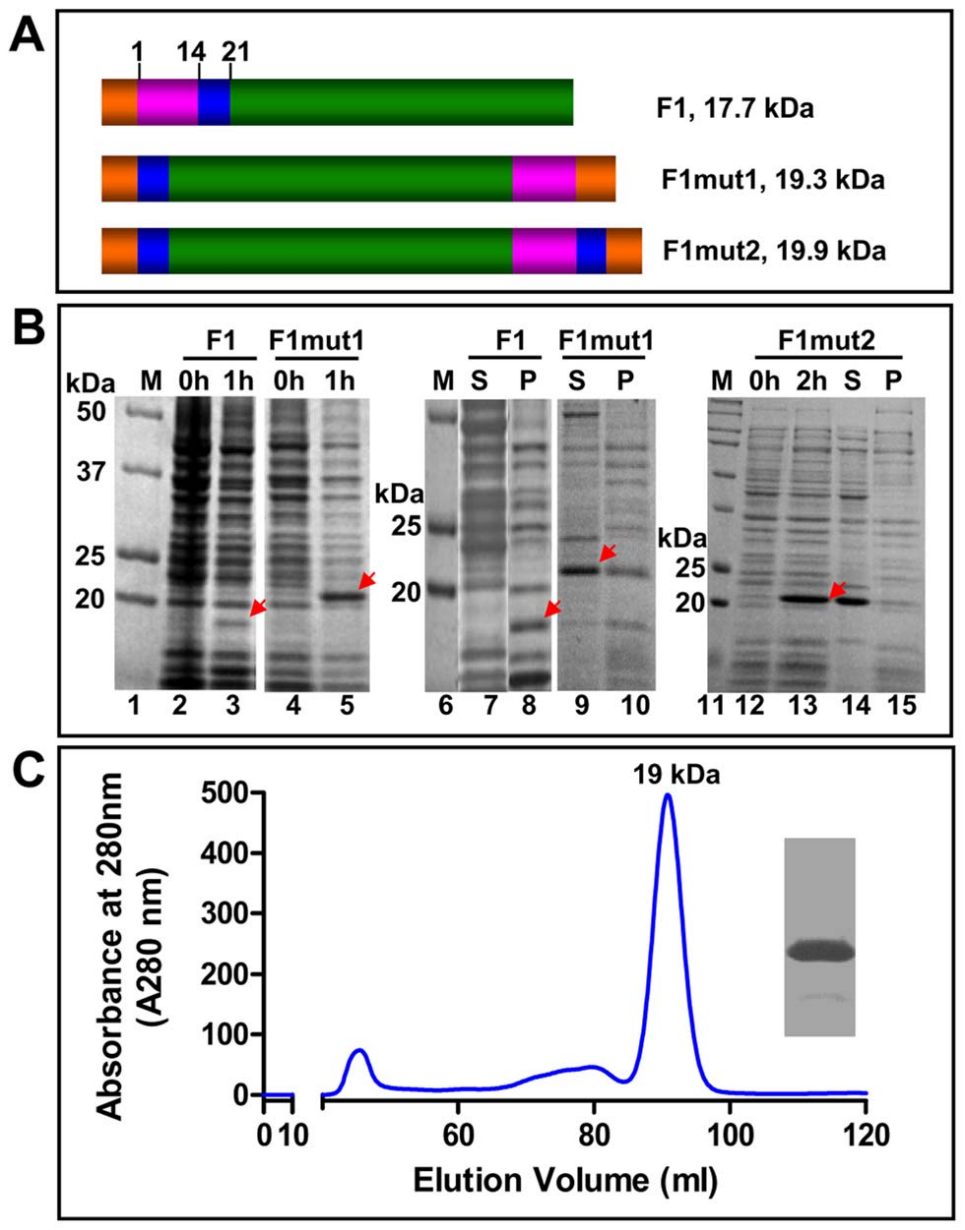
Designing monomeric F1 mutants. (**A**) Schematic of native F1, F1mut1, and F1mut2 recombinant constructs. The donor β-strand of F1 is shown in pink, the T cell epitope region in blue, and the rest of the F1 coding sequence in green. The numbers correspond to the aa residues of F1. Native F1 has one hexa-histidine tag (orange) at the NH_2_-terminus, whereas F1mut1 and F1mut2 have two hexa-histidine tags, one at the NH_2_-terminus and another at the COOH-terminus. (**B**) Expression and solubility analysis. The recombinant F1 proteins were over-expressed by adding IPTG to 1 mM final concentration. The samples at 0, 1, or 2 h time points were analyzed by SDS-PAGE (15% gel) and Coomassie blue staining. The positions of F1 protein bands are marked with red arrows. The samples at 1 h or 2 h time points were analyzed for solubility using the B-PER reagent. S, soluble fraction (supernatant from 12,000 *g* centrifugation of the lysate); P, insoluble fraction (pellet); M, molecular weight standards. (**C**) Purification of F1mut1. The F1mut1 recombinant protein was purified from the cell-free lysates by HisTrap affinity chromatography followed by Hi-load 16/60 Superdex 200 gel filtration. The molecular weight of F1mut1 peak fraction was calculated from the calibration curve constructed by gel filtration on the same column of standard proteins of known molecular weight [Thyroglobulin (669 kDa), Ferritin (440 kDa), Catalase (232 kDa), aldolase (158 kDa), Ovalbumin (43 kDa), RNase A (14 kDa), and Albumin (67 kDa)]. The insert shows the purity of F1mut1 protein after SDS-PAGE and Coomassie blue staining of the peak fraction. Similar results were obtained with the F1mut2 recombinant protein. See Materials and Methods for additional details.

The X-ray structure and biochemical studies established that F1 polymerizes into a linear fiber by head to tail interlocking of F1 subunits through a donor strand complementation mechanism [10] (Figure 1B). Each subunit has an Ig-like domain consisting of a four-stranded anti-parallel β-sheet. Of the four β-strands, three belong to one subunit forming a cleft into which the NH_2_-terminal β-strand of the “n+1” subunit locks in, resulting in a bridge connecting adjacent subunits (inter-molecular complementation) (Figure 1B). Stringing of subunits in this fashion leads to assembly of linear F1 fibers of varying lengths. Caf1M chaperone is required for this process because prior to filling the cleft, a “spare” β-strand of Caf1M temporarily occupies the cleft until it is replaced by the β-strand of the incoming subunit with the assistance of an outer membrane *usher* protein, Caf1A. Over-expression of the F1 gene in a heterologous system such as *E. coli* (Figure 2) exposes the unfilled hydrophobic cleft, resulting in uncontrolled aggregation of F1 subunits into insoluble inclusion bodies. This is demonstrated in Figure 2B in which all of the over-produced F1 protein partitioned into the pellet (lane 8) and none was detected in the supernatant (lane 7). Denaturation of the insoluble protein recovered some of the F1 protein into the soluble fraction but it still aggregated rapidly leading to precipitation (in the Histrap column) upon removal of the denaturant. Similar aggregation behavior of F1 was observed in previously published studies [38], [39].

We hypothesized that shifting of the NH_2_-terminal β-strand of F1 to the COOH-terminus should reorient the β-strand such that it fills its own cleft (intra-molecular complementation (Figure 1B), and furthermore, it should no longer require the assistance of chaperone or *usher* proteins. To test this hypothesis, we constructed an F1 mutant (F1mut1) by deleting the NH_2_-terminal donor strand [amino acid (aa) residues 1–14] and fusing it to the COOH-terminus with a short (Serine-Alanine) linker in between (Figure 2A). The recombinant F1mut1, as predicted, folded into a soluble protein in the absence of Caf1M or Caf1A, and approximately 70% of the protein partitioned into the cell-free lysate (Figure 2B, lanes 9 and 10). In addition, for reasons unknown, the mutated F1 protein was expressed at significantly higher levels than that of the native F1 protein after IPTG induction (Figure 2B, compare lane 5 with lane 3). The gel filtration profile showed that the F1mut1 eluted as a symmetrical peak corresponding to a molecular mass of ∼19 kDa (Figure 2C), a monomer, suggesting that the interlocking mechanism had shifted from inter- to intra-molecular interactions.

### Restoring the potential T cell epitopes of F1 mutant

A bioinformatics approach was used to determine if the strand shifting might have disrupted the NH_2_-terminal epitopes of F1. The aa residues 7 to 20 are reported to contain a mouse H-2-IA*^d^* restricted CD4^+^ T cell epitope [40]. Of the fifty-three predicted 9-mer CD8^+^ T cell epitopes that encompassed 46 human MHC-I alleles (Table S1 in Text S1), four peptides (aa residues: 9–17, 10–18, 11–19 and 13–21) fell in this region, and of the 9 peptides predicted to contain CD4^+^ T cell epitopes (Table S2 in Text S1), only one (aa residues 1–18) belonged to this region. We determined that the integrity of these potential linear epitopes could be restored by extending the sequence of the switched strand by up to the aa residue 21, which would duplicate the residues 15 to 21 at the COOH-terminus. Thus, the F1mut2 was constructed (Figure 2A) and tested. The F1mut2 behaved in a similar manner as the F1mut1 with respect to over-production and solubility (Figure 2B, lanes 12–15) and was also purified as a monomer (data not shown).

### Construction of mutated F1-V immunogens

**Figure 3:**
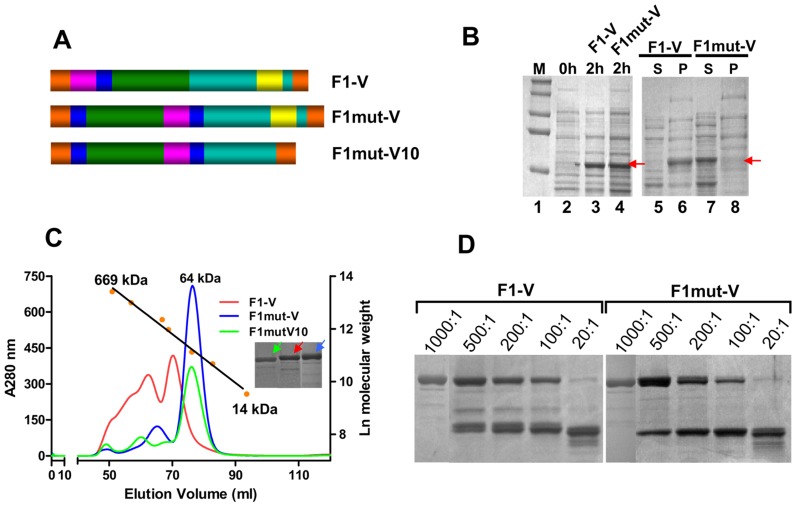
Construction of mutated F1-V immunogens. (**A**) Schematic of native F1-V, F1mut-V and F1mut-V10. Cyan represents the coding sequence of V antigen, and yellow, the putative immunomodulatory sequence that is part of V sequence. Rest of the colors represents the same as described in legend to Figure 2. (**B**) Expression and solubility analysis of F1-V constructs were performed using the B-PER reagent. The samples were analyzed by SDS-PAGE and Coomassie blue staining. The positions of the F1-V protein bands are marked with red arrows. S, soluble fraction (supernatant from 12,000 *g* centrifugation of the lysate); P, insoluble fraction (pellet); M, molecular weight standards. (**C**) F1-V, F1mut-V and F1mut-V10 were purified by HisTrap column chromatography followed by Hi-load 16/60 Superdex 200 gel filtration. The calibration graph was generated by passing various molecular weight standards through the same column [Thyroglobulin (669 kDa), Ferritin (440 kDa), Catalase (232 kDa), aldolase (158 kDa), Ovalbumin (43 kDa), RNase A (14 kDa), and Albumin (67 kDa)]. The insert shows the purity of F1-V, F1mut-V, and F1mut-V10 proteins following SDS-PAGE and Coomassie blue staining of the peak fractions. The color of arrows corresponds to the color of the elution profiles of various proteins. (**D**) Stability of F1-V and F1mut-V proteins was tested by treatment with increasing amounts of trypsin at room temperature overnight. The ratios shown above the gel correspond to the ratios of F1-V or F1mut-V proteins to trypsin (wt∶wt). See Materials and Methods for additional details.

Fusion of F1mut2 to V would generate a bivalent plague vaccine. Consequently, a mutated F1-V fusion protein (F1mut-V) was produced by fusing F1mut2 to the NH_2_-terminus of V with a two aa linker in between (Figure 3A), and its solubility was compared to that of the native polymeric F1-V. The native F1-V protein, as reported previously [20], [22], was insoluble and partitioned into inclusion bodies (Figure 3B; lanes 5 and 6). Denaturation and refolding solubilized some of the protein but it also eluted, as was reported previously [20], over a wide range of high molecular weight sizes in a gel filtration column (Figure 3C, red profile). F1mut-V protein, on the other hand, was nearly 100% soluble (Figure 3B; lanes 7 and 8) and eluted as a symmetrical peak corresponding to a molecular weight of ∼64 kDa, equivalent to the mass of monomeric F1mut-V fusion protein (Figure 3C, blue profile). The yield of F1mut-V was quite high, ∼20 mg pure protein per liter of the *E. coli* culture. Furthermore, its stability to trypsin digestion was similar to that of the native F1-V (Figure 3D).

The *Y. pestis* V antigen has been reported to induce interleukin (IL)-10 and suppress the production of pro-inflammatory cytokines such as interferon (IFN)-γ and tumor necrosis factor (TNF)-α, which could lead to lowering of innate immunity in vaccinated individuals [41]. A truncated V in which the COOH-terminal 30 aa residues (271–300) were deleted (referred to as “V10” mutation) was reported to lack this immunomodulatory function [41]. A mutated F1mut-V10 recombinant was therefore constructed by deleting these residues (Figure 3A). This mutant protein was also over-produced in *E. coli*, which was also highly soluble and could be purified as a monomer (Figure 3C, green profile).

### Designing an oligomerization deficient YscF mutant

**Figure 4:**
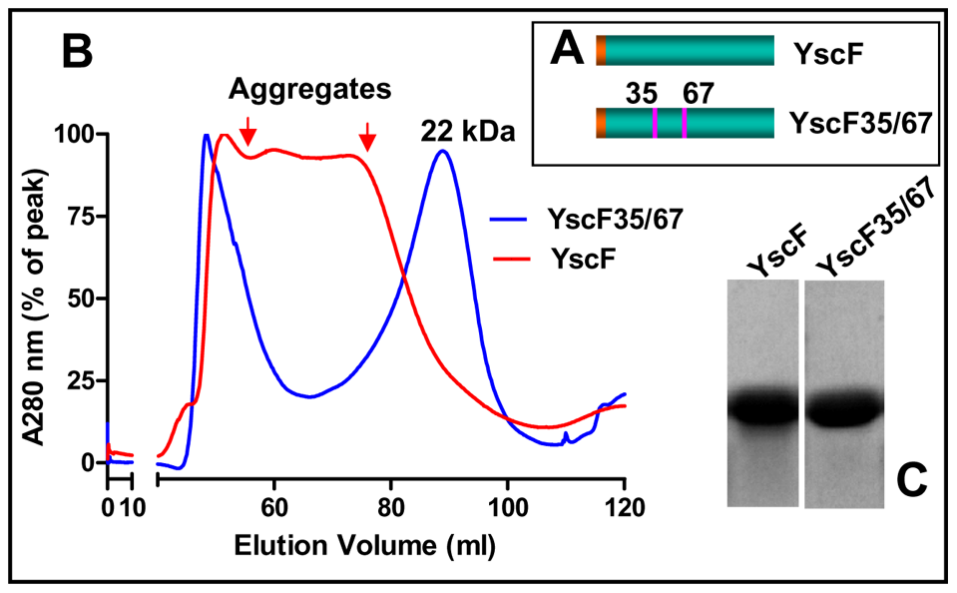
An oligomerization deficient YscF mutant. (**A**) Schematic of native YscF and YscF35/67 mutants. (**B**) Purification of YscF and YscF35/67 mutant proteins. The gel filtration profiles showed that the native YscF eluted as a broad peak spanning the entire high molecular weight range and the mutated YscF35/67 eluted as two peaks, one as a high molecular weight aggregate near the void volume, and another at 22 kDa corresponding to the size of a dimer. (**C**) Purity of YscF and YscF35/67 proteins as analyzed by SDS-PAGE and Coomassie blue staining of the peak fractions.

Inclusion of YscF might expand the breadth of efficacy of F1-V plague vaccine formulation to *Y. pestis* strains containing variant V antigens [26], or of those strains devoid of capsule but highly virulent in nature [24], [25]. Since YscF is a structural component of the injectisome, over-production of this protein caused aggregation [42]. A mutant YscF was constructed by mutating the aa residues Asn35 and Ile67, that are involved in oligomerization (Asn35 changed to Ser, and Ile67 changed to Thr) (Figure 4A) [43]. The resultant YscF35/67 mutant protein was soluble and the gel filtration profile showed two peaks, a high molecular weight aggregate near the void volume, and a second peak corresponding to a molecular mass of ∼22 kDa, which is equivalent to a dimer (Figure 4B, blue profile; C). The native YscF, on the other hand, eluted over a wide range of high molecular weight sizes consistent with the formation of heterodisperse aggregates (Figure 4B, red profile). The mutant dimer did, however, show slow aggregation during concentration and storage, as evident by the appearance of small amounts of precipitates.

### Decorating the phage T4 nanoparticle with F1 and V antigens

**Figure 5:**
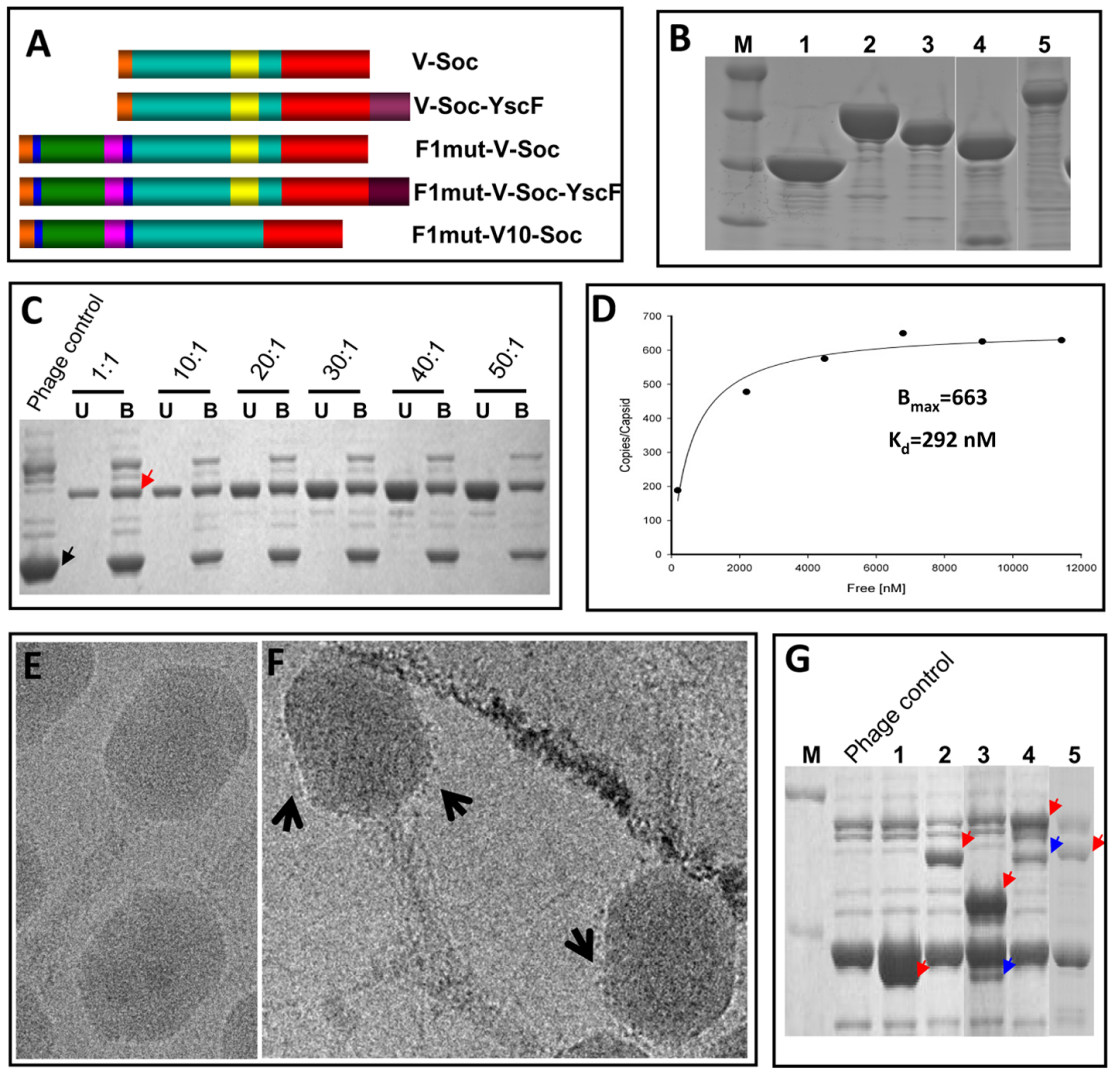
Engineering of F1, V, and YscF antigens and display on phage T4 nanoparticle. (**A**) Schematic of Soc-fusions. Soc is shown in red and YscF in brown. Rest of the colors represents the same as shown in Figures 2 and 3. V-Soc, V was fused to the NH_2_-terminus of Soc; V-Soc-YscF, V was fused to the NH_2_-terminus and YscF to the COOH-terminus of Soc; F1mut-V-Soc, F1mut-V was fused to the NH_2_-terminus of Soc; F1mut-V-Soc-YscF, YscF was fused to the COOH-terminus of F1mut-V-Soc; F1mut-V10-Soc, F1mut-V10 was fused to the NH_2_-terminus of Soc. (**B**) The Soc fusion proteins in panel A were over-expressed and purified as described in Materials and Methods. The purity of the proteins was evaluated by SDS-PAGE and Coomassie blue staining. Lanes: M, molecular weight standards; 1, V-Soc; 2, F1mut-V-Soc; 3, F1mut-V10-Soc; 4, V-Soc-YscF; 5, F1mut-V-Soc-YscF. (**C**) Display of F1mut-V-Soc on phage T4. Approximately 3×10^10^ Hoc^−^Soc^−^ phage particles were incubated at the indicated ratios of F1mut-V-Soc molecules to capsid binding sites and display was carried out as described in Materials and Methods. Lanes: Phage control, Hoc^−^ Soc^−^ phage used in the experiment (the position of gp23* band is labeled with a black arrow); U and B represent the unbound and phage-bound fractions. See the appearance of F1mut-V-Soc band in the bound lanes (red arrow), which is not present in the phage control. (**D**) Saturation binding curve of F1mut-V-Soc. The density volumes of bound and unbound proteins from SDS-PAGE (**C**) were determined by laser densitometry and normalized to that of gp23* present in the respective lane. The copy numbers were determined in reference to gp23* (930 copies per capsid). The data were plotted as one site saturation ligand binding curve and fitted by non-linear regression using the SigmaPlot10.0 software and the calculated binding parameters are shown. K_d_, apparent binding constant; B_max_, maximum copy number per phage particle. (**E and F**) Cryo-electron micrograph of wild-type control phage T4 (E) and phage T4 decorated with F1mut-V (F). Arrows point to a layer of fuzzy projections around the perimeter of the capsid in the F1mut-V decorated phage. (**G**) Various Soc fusion proteins displayed on phage T4 for immunizations. Lanes: M, molecular weight standards; Phage control, Hoc^−^ Soc^−^ phage used in the experiment; 1, V-Soc; 2, F1mut-V-Soc; 3, V-Soc-YscF; 4, F1mut-V-Soc-YscF; 5, F1mut-V10-Soc. Red arrows show the positions of various displayed protein bands. Presence of a second fainter and shorter band in lanes 3 and 4 (blue arrows) indicate that some of the C-terminally fused YscF was cleaved off by nonspecific proteolysis.

A large number of F1, V, F1-V, and YscF recombinant proteins, both in native and mutated forms, were fused to the NH_2_- and/or the COOH-termini of either phage T4 Soc or the T4-related phage RB69 Soc and screened for their solubility as well as ability to bind to T4 phage (Figure 5A, and data not shown). Our previous studies showed that the RB69 Soc binds to T4 capsid at nearly the same affinity as T4 Soc [34]. The RB69 Soc-fused plague antigens, with the exception of the native F1-Soc, produced soluble proteins whereas the T4 Soc-fused antigens were insoluble. Several of the RB69 immunogens were purified (Figure 5B) and tested for binding to T4 using our previously established *in vitro* assembly system. A typical result is shown in Figure 5C and D, which also exemplifies the versatility of the T4 nanoparticle display. Consistent with the crystal structure of Soc, which showed that both the NH_2_- and COOH-termini are exposed on the capsid surface, the plague immunogens F1mut and V could be efficiently displayed as an F1mut-V fusion protein that in turn was fused to the NH_2_-terminus of Soc (Figure 5C). At the same time, its COOH-terminus could be fused to YscF35/67, and the resultant F1mut-V-Soc-YscF35/67 fusion protein containing all three plague immunogens could be displayed on T4 capsid (Figure 5G, lane 4).

The 66 kDa F1mut-V-Soc bound to T4 even at a relatively low 1∶1 ratio of F1mut-V-Soc molecules to Soc binding sites (Figure 5C, red arrow). Binding increased with increasing ratio and reached saturation at 20–30∶1. The copy number of bound F1mut-V-Soc per capsid (B_max_) was 663, which meant that ∼76% of the Soc binding sites were occupied, and its apparent binding affinity (K_d_) was 292 nM, which was ∼4-fold lower than that of Soc binding (K_d_ = 75 nM) [34] (Figure 5D). This is consistent with the expectation that the 66 kDa F1mut-V-Soc, unlike the 10 kDa Soc, would encounter steric constraints to occupy all the binding sites on the capsid exterior. Given this limitation, the observed copy number was remarkably high, with the capsid surface presumably tightly packed with the F1mut-V molecules (model shown in Figure 1F) and exposing, consequently, the plague antigen epitopes for presentation to the immune system. Indeed, cryo-electron microscopy showed that these T4 capsids, unlike the wild-type capsids (Figure 5E), were decorated with a layer of F1mut-V molecules, seen as fuzzy protrusions around the perimeter of the capsid wall (Figure 5F). A series of nanoparticle decorated plague immunogens were prepared, including all three plague immunogens displayed on the same capsid using the F1mut-V-Soc-YscF35/67 fusion protein (Figure 5G, lane 4).

### The mutated F1-V monomer induced robust immunogenicity and protective efficacy

**Figure 6:**
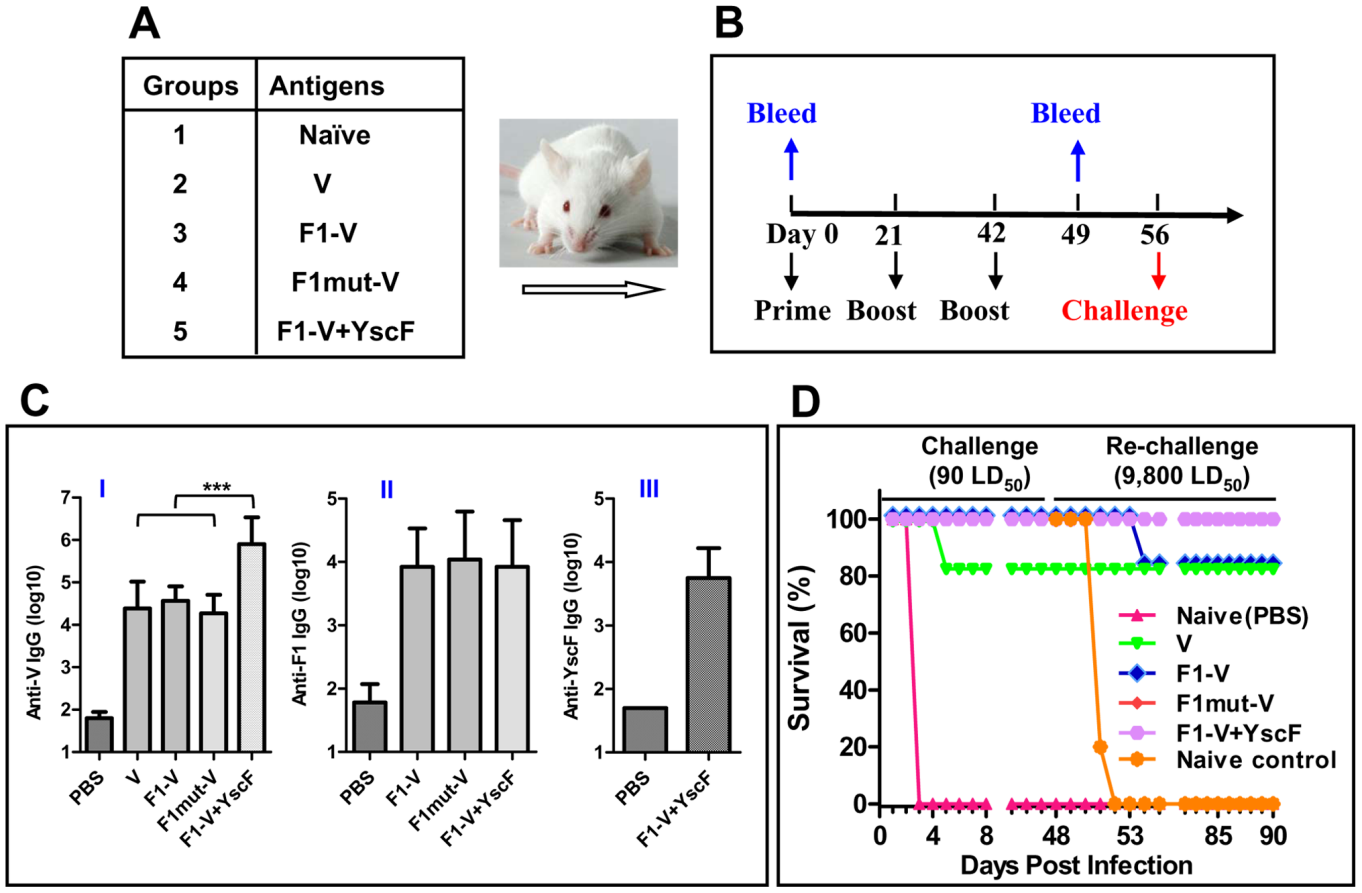
The soluble monomeric F1 mutant protein elicits robust antibody titers and provides complete protection in a mouse model of pneumonic plague. The immunogenicity and protective efficacy of F1mut-V and other plague immunogens were evaluated in a mouse model. (**A**) Balb/c mice, twelve per group, were vaccinated with various plague antigens adjuvanted with alhydrogel. (**B**) Immunization scheme. (**C**) Antigen-specific antibody (IgG) titers were determined by ELISA, using purified V (I), F1mut2 (II), or YscF35/67 (III) as the coating antigen. No significant cross-reactivity was observed between the antibodies produced against one plague antigen versus a different plague antigen that was coated on the ELISA plate. Error bars represent S.D. “***” denotes p<0.001 (ANOVA). (**D**) Survival of immunized mice against intranasal challenge with 90 LD_50_ of *Y. pestis* CO92. The survived mice were re-challenged with 9,800 LD_50_ at day-48 post-first challenge. See Materials and Methods for additional details. The animal mortality data was analyzed by Kaplan Meier's survival estimates and a p value of ≤0.05 was considered significant.

The immunogenicity of mutated F1 immunogen was tested in a mouse model. The animals (Balb/c) were immunized according to the scheme shown in Figure 6 A and B and antibody titers in the sera were determined by ELISA. The data showed that all the three plague antigens adjuvanted with alhydrogel induced antigen-specific antibodies (Figure 6C). The V antigen induced the highest titers with the end point titer reaching as high as 7×10^6^. The YscF antigen was the least immunogenic (Figure 6C, panel III), with the endpoint titers about 1–2 orders of magnitude lower than that of F1 and V antigens (Figure 6C, panels I and II). No significant differences in F1-specific antibody titers were observed among the various groups (i.e., F1-V versus F1mut-V versus F1-V+YscF; panel II). Importantly, the monomeric F1mut-V induced comparable antibody titers as the native polymeric F1-V, suggesting that the capsular structure of F1 *per se* did not afford a significant advantage to induction of antibodies. However, unexpectedly, the V-specific IgG titers were at least an order of magnitude higher when YscF was also included in the vaccine (p<0.001) (Figure 6C, panel I; compare F1-V to F1-V+YscF).

Intranasal challenge of animals with 90 LD_50_ of *Y. pestis* CO92 [1 LD_50_ = 100 colony forming units (CFU) in Balb/c mice], one of the most lethal strains, showed that all the control mice died by day 3. However, the mice immunized with native V immunogen showed 83% survival (two of twelve mice died), whereas the mice immunized with F1-V, F1mut-V, or F1-V plus YscF were 100% protected (Figure 6D). The surviving mice were then re-challenged with a much higher dose, 9,800 LD_50_, of *Y. pestis* CO92 on day-48 post-first challenge. The purpose of re-challenge was to determine if a strong adaptive immunity was generated after first infection with *Y. pestis*, which should in turn confer a much higher level of protection against subsequent challenges. Indeed, our data showed that all of the mice survived the re-challenge except two mice in the native F1-V group that succumbed to infection (83% protection) (Figure 6D). All of the naïve animals of same age which were used as a re-challenge control died as expected. These efficacy results showed that the monomeric F1mut-V was as efficacious as or even slightly better than the native F1-V polymer.

### The T4 nanoparticle arrayed antigens provided complete protection against *Y. pestis* pneumonic challenge

**Figure 7:**
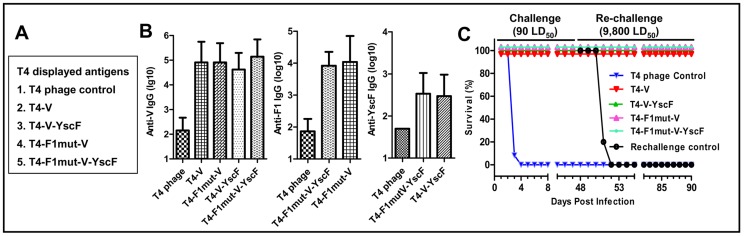
The T4 nanoparticle displayed plague immunogens induced robust immunogenicity and protective efficacy against pneumonic plague. The immunogenicity and protective efficacy of T4 displayed plague immunogens were evaluated in a mouse model using the same immunization scheme shown in Figure 6B. (**A**) The T4 displayed plague immunogen groups, twelve mice per group. The Soc-fused plague immunogens were displayed on T4 phage particles and were directly used for vaccination without any adjuvant. (**B**) Antigen-specific antibody (IgG) titers as determined by ELISA. (**C**) Survival of vaccinated mice against intranasal challenge with 90 LD_50_ of *Y. pestis* CO92. The survived mice were re-challenged with 9,800 LD_50_ at day-48 post-first challenge. The animal mortality data was analyzed by Kaplan Meier's survival estimates and a p value of ≤0.05 was considered significant.

The immunogenicity of nanoparticle decorated plague antigens was tested by vaccinating mice with phage T4 particles (Figure 7A). The amount of the antigen was kept the same as that of the soluble preparations (Figure 6); however, the T4 formulations contained no adjuvant. The data showed that the T4 displayed plague antigens induced comparable antibody titers as the adjuvanted soluble antigens (Figure 7B). The challenge data showed that all the T4 decorated plague antigens, including the V alone group, provided 100% protection to mice against intranasal challenge with 90 LD_50_ of *Y. pestis* CO92; all the control animals died by day 4. Upon re-challenge on day 48 post-first challenge with 9,800 LD_50_ (Figure 7C), all of the mice were completely protected. As expected, the control re-challenge group of mice succumbed to infection. Overall, these data suggested that the T4 nanoparticle arrayed plague antigens might be more potent than the soluble antigens, as two deaths in each of the V and F1-V groups of mice occurred with the soluble vaccines (Figure 6D) but not with the T4 vaccines.

### The T4 nanoparticle antigens induced balanced T_H_1 and T_H_2 immune response

**Figure 8:**
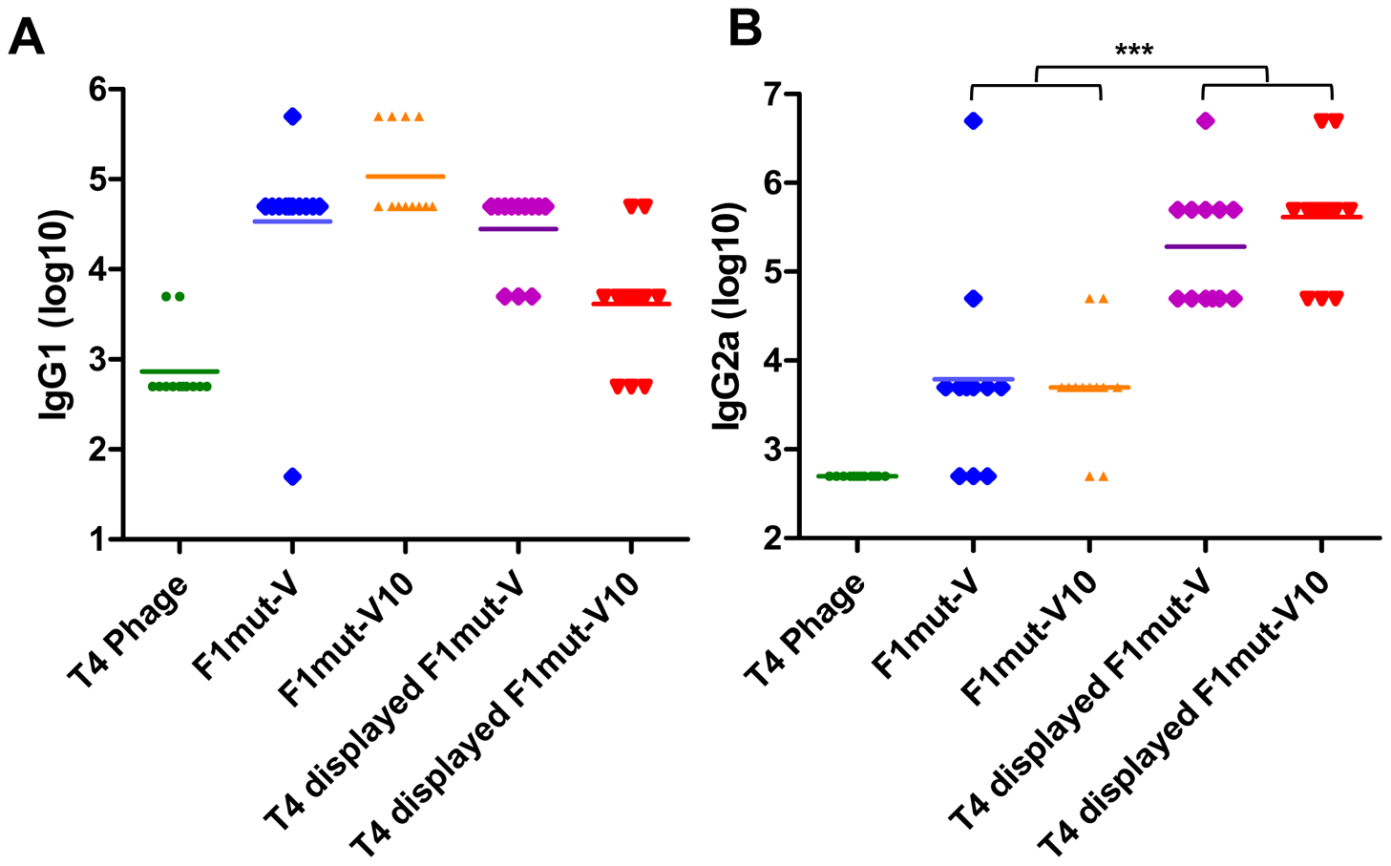
The T4 displayed plague immunogens generated balanced T_H_1 (**IgG1) and T_H_2 (IgG2a) responses.** The immunization scheme is shown in Figure 6B. Seven days after the second boost, sera were collected and IgG1 (**A**) and IgG2a (**B**) titers were determined by ELISA. F1mut-V was used as the coating antigen, since it covers all the epitopes present in both F1mut-V and F1mut-V10. Note that the sera of the control T4 phage-immunized mice showed higher background. This was because T4 phage, as demonstrated in previous studies, induces a strong antibody response to its components. Consequently, the sera from T4 phage- immunized mice will have increased amounts of IgGs compared to the pre-immune sera, giving more non-specific background at low dilutions of the sera. Data shown are the antibody titers of 12 mice in each group with S.D. (error bars). *, p<0.05; ***, p<0.001 (ANOVA).

Stimulation of both arms of the immune system, humoral (T_H_2) and cellular (T_H_1), is probably essential for protection against *Y. pestis* infection [6], [23], [44], [45]. In mice, the T_H_1 profile involves induction of antibodies belonging to IgG2a subclass whereas the T_H_2 profile primarily involves the induction of IgG1 subclass. To determine the specificity of antibodies induced by soluble *vs* T4 displayed antigens, the subclass IgG titers were determined by ELISA (Figure 8). These data showed that the soluble antigens and the T4 displayed antigens induced comparable IgG1 titers (T_H_2 response) (Figure 8A) whereas the T4 antigens evoked 1–2 orders of magnitude higher IgG2a titers than the soluble antigens (T_H_1 response) (Figure 8B). These results suggested that the T4 decorated plague immunogens stimulated stronger cellular responses as well as humoral responses, whereas the soluble antigens showed a bias towards the humoral responses as was observed in the previous studies [46].

### F1mut-V and F1mut-V10 showed similar immunogenicity and protective efficacy profiles

**Figure 9:**
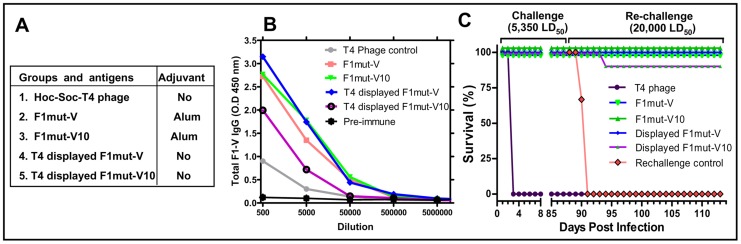
The F1mut-V and F1mut-V10 mutants show comparable immunogenicity and protection against pneumonic plague. The immunogenicity and protective efficacy of F1mut-V and F1mut-V10 were compared both as adjuvanted soluble antigens or adjuvant-free T4 nanoparticle decorated antigens. (**A**) The vaccine formulations used in the study, eight mice per group. (**B**) Total F1-V specific antibody titers as determined by ELISA. Note that the sera of the control T4 phage-immunized mice showed higher background than the pre-immune sera, probably because T4 phage induces a strong antibody response to its components which raises the levels of the IgGs in the sera and gives more non-specific background at low dilutions. (**C**) Survival of vaccinated mice against intranasal challenge with 5,350 LD_50_ of *Y. pestis* CO92. The survived mice were re-challenged with 20,000 LD_50_ at day-88 post-first challenge. The animal mortality data was analyzed by Kaplan Meier's survival estimates and a p value of ≤0.05 was considered significant.

**Figure 10:**
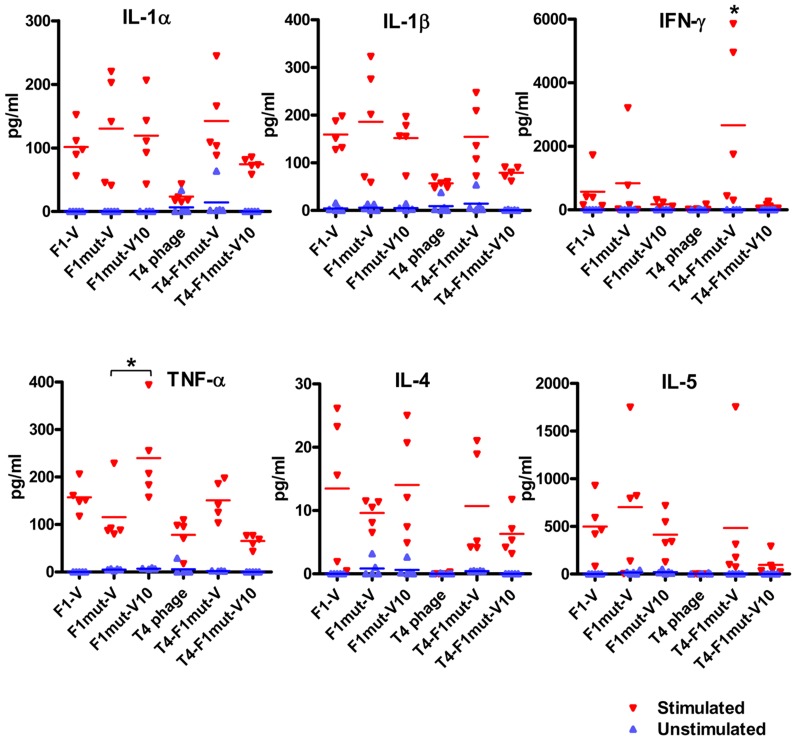
Induction of proinflammatory cytokines by F1mut-V and F1mut-V10 immunogens. Seven days after the second boost (day-49), mice (5 per group) were sacrificed and spleens were harvested. The splenocytes were cultured and stimulated by purified F1-V protein. Cytokines levels were determined as described in Materials and Methods.

The immunogenicity and protective efficacy of F1mut-V *vs* F1mut-V10 was evaluated by three criteria: F1- and V-specific antibody titers, cytokine responses, and protection against *Y. pestis* CO92 challenge. Both the F1- and V-specific IgG antibodies (Figure 9B) and subclass IgG titers (Figure 8A and B) were not significantly different between the F1mut-V and F1mut-V10 immunized groups of mice when the immunogen used was soluble and alhydrogel-adjuvanted. However, when decorated on phage T4 nanoparticle with no adjuvant, F1mutV elicited higher total IgG (Figure 9B) and IgG1 titers (Figure 8A) than F1mutV10 (p<0.05). These trends were also reflected in the production of the T_H_2 cytokines, IL-4 and IL-5, by splenocytes of immunized mice stimulated *ex vivo* with F1-V. Similar levels of IL-4 and IL-5 were produced by the soluble F1mut-V and F1mut-V10 antigens or the T4-displayed F1mut-V, whereas the T4 displayed F1mut-V10 showed slightly reduced levels (Figure 10). The induction of proinflammatory cytokines, such as IL-1α and IL-1β was also similar, irrespective of whether the antigens were soluble or T4 displayed (Figure 10). However the levels of TNF-α, an inflammatory mediator that synergistically acts with IFN-γ to help bridge the gap between innate and cell-mediated immune responses, were significantly higher in mice immunized with soluble F1mut-V10 than those immunized with F1mut-V (Figure 10). However, the trend was opposite when F1mut-V and F1mut-V10 immunogens were T4 displayed, although the data did not reach statistical significance. In fact, the T4 displayed F1mut-V10 induced overall weaker IFN-γ and cytokine responses when compared to its F1mut-V counterpart.

With respect to animal survival, both the F1mut-V and F1mut-V10 immunogens, either soluble or T4 displayed, provided 100% protection to mice upon intranasal challenge with 5,350 LD_50_ of *Y. pestis* CO92 (Figure 9C), with the control animals dying by day 3. When the mice were re-challenged with an extremely high LD_50_ (20,000) on day 88 post-first challenge, all the groups showed 100% protection except the T4-displayed F1mut-V10 group in which one mouse died (92% protection) (Figure 9C). All of the naïve re-challenge control animals died by day 4.

### The mutated and T4 displayed plague antigens provided 100% protection against pneumonic plague in a Brown Norway rat model

**Figure 11:**
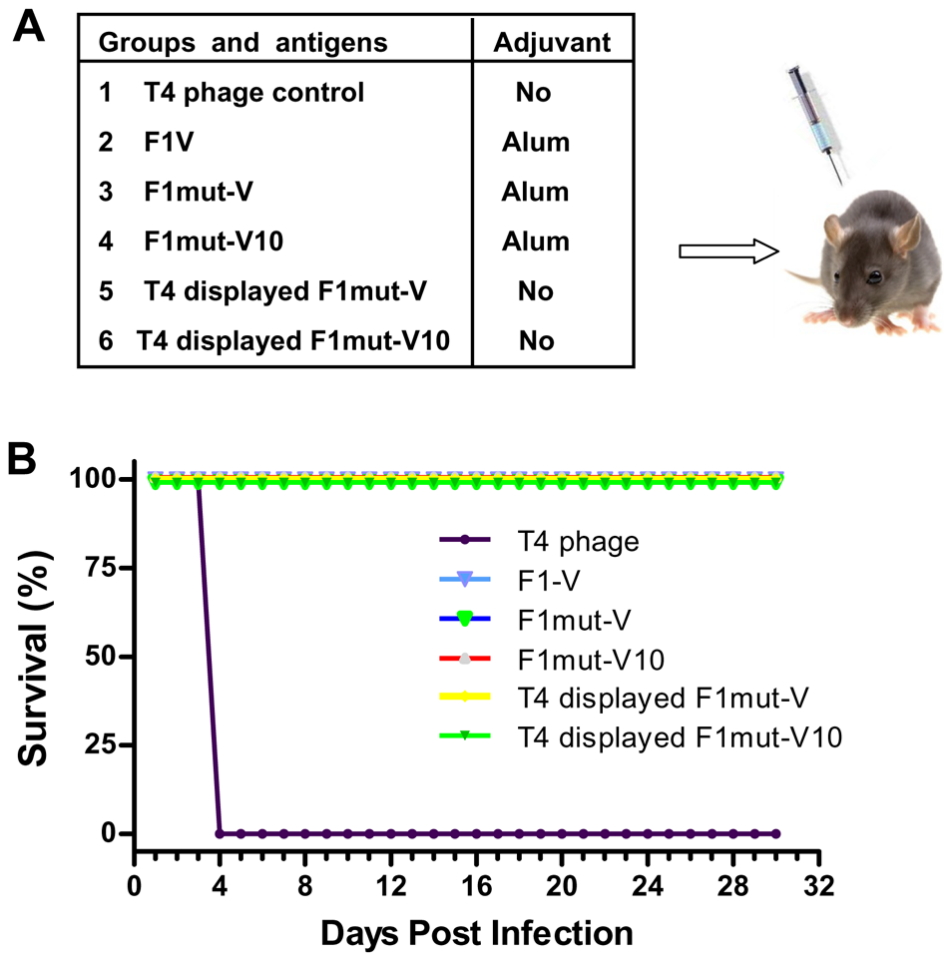
The mutated and T4 displayed plague antigens provided complete protection against *Y. pestis* CO92 in a Brown Norway rat model of pneumonic plague. (**A**) Vaccine formulations used in various groups, twelve rats per group. The rats were immunized as per the basic scheme shown in Figure 6. The soluble antigens (groups 2–4) were adjuvanted with alhydrogel. The T4 displayed groups contained no adjuvant. (**B**) Survival of vaccinated rats against intranasal challenge with 5,000 LD_50_ of *Y. pestis* CO92. The animal mortality data was analyzed by Kaplan Meier's survival estimates and a p value of ≤0.05 was considered significant.

To further test the efficacy of the mutated inmunogens, a rat study was conducted. Rats [47], the natural host of *Y. pestis*, were vaccinated with alhydrogel adjuvanted F1mut-V, and F1mut-V10 as well as the T4 nanoparticle displayed F1mut-V and F1mut-V10 (Figure 11A). The same immunization scheme as shown in Figure 6B was used and the animals were challenged with a 5,000 LD_50_ of *Y. pestis* CO92. The data showed that all the control animals died by day 4 whereas all the F1mut-V and F1mut-V10 immunized animals were 100% protected (Figure 11B).

## Discussion

Since the deadly anthrax attacks in 2001, stockpiling of recombinant anthrax and plague vaccines to protect masses against a potential bioterror attack became a national priority. However, no plague vaccine has yet been licensed. The reasons include poor stability, insufficient immunogenicity, and/or manufacturing difficulties associated with the current formulations. New immunogen designs and vaccine platforms that could overcome some of these problems would be of great interest not only to stockpile efficacious biodefense vaccines but also to develop vaccines against a series of infectious diseases of public health importance. Here, by using structure-based immunogen design and T4 nanoparticle delivery approaches, we have engineered new and efficacious plague vaccines that could be manufactured relatively easily and provide complete protection against pneumonic plague in two rodent models.

The surface-exposed *Y. pestis* antigens F1 and V have been the leading candidates for formulating a subunit plague vaccine for nearly two decades [14], [15], [16], [17]. Although poorly immunogenic by themselves, their immunogenicity could be enhanced by adjuvantation with Alum [15] or by fusion with a molecular adjuvant such as flagellin [19]. While complete protection was observed in rodent models [17], these vaccines impart partial and varied protection in African Green monkeys [6], [17]. Another concern has been that the naturally polymeric F1 has high propensity to aggregate (Figure 2). When produced in a heterologous system such as *E. coli*, the recombinant F1-V protein partitions into insoluble inclusion bodies [15], [19], [20], [21], [22] (Figure 3). Although it can be partially recovered in soluble form by denaturation and re-folding, the preparation still consists of a mixture of heterogenous aggregates and varying amounts of the misfolded protein [20]. These might also trap contaminants, compromising the overall purity, stability, and efficacy of the vaccine. Attempts to produce a monomeric vaccine by mutating the lone cysteine residue in V have not been successful [20].

We proposed three hypotheses to design a soluble monomeric plague vaccine, yet retaining its structural and epitope integrity. First, we hypothesized that the β-strand that connects the adjacent F1 subunits requires repositioning. This was achieved by transplanting the NH_2_-terminal β-strand to the COOH-terminus in such a way that the reoriented β-strand fitted into its own β-sheet cleft rather than that of the adjacent F1 subunit. It also eliminated the need for chaperone and *usher* mediated oligomerization as there would no longer be an unfilled β-sheet pocket exposed in the F1 subunit. Second, by using epitope predictions, the NH_2_-terminal aa residues 15–21 of F1 flanking the β-strand were duplicated at the COOH-terminal end to restore any potential T-cell epitopes that might have been lost during the switch. This is important because in a previous study, a simple β-strand switch produced a less stable monomer with diminished immunogenicity [48]. Third, the mutated F1 was fused to the NH_2_-terminus of V with a flexible linker in between to minimize interference between the F1 and V domains. The bivalent F1mut-V immunogen thus produced showed a remarkable shift in solubility, from an insoluble F1-V polymer to a completely soluble monomer (Figure 3). The monomer could be purified from cell-free lysates at high yields, ∼20 mg of pure protein from a liter of *E. coli* culture, which we believe could be substantially increased under optimized conditions in a fermentor.

Several lines of evidence demonstrated that the F1mut-V monomer was as efficacious as, if not better than, the native F1-V polymer. In four separate immunization studies and two animal models (Figures 6, 7, 9, and 11), F1mut-V induced robust immunogenicity and protective efficacy. It showed similar levels of F1- and V-specific antibody titers as the native F1-V, and no significant differences were observed in T_H_1 vs T_H_2 specific IgG subclass titers. Furthermore, F1mut-V overall showed stronger cytokine responses and conferred 100% protection in vaccinated mice and rats, including when very high doses of *Y. pestis* CO92, ∼5,350 LD_50_ for first challenge and ∼20,000 LD_50_ for re-challenge, were administered by the intranasal route (Figure 9). The native F1-V, on the other hand, showed slightly lower protection (∼83%) upon re-challenge (Figure 6).

The possibility of increasing the breadth and potency of F1-V vaccine by inclusion of YscF was tested by constructing an oligomerization deficient YscF35/67 mutant [43]. Such a vaccine might be effective even against those *Y. pestis* strains that contain variant V antigens or lack the capsule, but are highly virulent [26]. The mutated protein, purified as a soluble dimer, elicited YscF-specific antibodies on its own, and, when it was mixed with F1-V, it enhanced the induction of V-specific antibody titers as well as survival rate in mice (Figure 6). While these results indicated enhanced potency of F1-V vaccine in the presence of YscF, more studies are needed to determine if the cost of an additional protein can be justified for vaccine manufacture. On the other hand, the T4 displayed trivalent vaccine, F1mut-V-Soc-YscF (Figures 5 and 7), might offer an alternative to incorporate YscF into the plague vaccine formulation.

*Y. pestis* infection stimulates IL-10 production which in turn suppresses the production of proinflammatory cytokines IFN-γ and TNF-α. Both IFN-γ and TNF-α are important for innate immunity, as well as to elicit T_H_1 immune responses that might be essential for protection against pneumonic plague [49], [50], [51]. These immunomodulatory functions, in part, were attributed to the V antigen, specifically to the NH_2_-terminal aa residues 31–49 [49]. Deletion of these residues, or of the COOH-terminal aa residues 271–300 (V10 mutation), have been reported to abrogate the suppression of IFN-γ and TNF-α [41], presumably by preventing the interaction of V with toll like receptor 2 (TLR2) and CD14, the receptors of the innate immune system [49], [52]. Our studies showed that both the F1mut-V and F1mut-V10 immunogens produced similar levels of IFN-γ and other proinflammatory cytokines, such as IL-1α and IL-1β, upon stimulation ex-vivo of splenocytes from immunized mice with F1mut-V. However, TNF-α was induced to significantly higher levels in the F1mut-V10 group (Figure 10), consistent with the published report [41]. However, the T4 nanoparticle decorated F1mut-V10 showed opposite trend, producing much reduced levels of TNF-α as well as IFN-γ and other cytokine responses than its F1mut-V counterpart, a result also correlated with lower protection against re-challenge [92% protection with T4 displayed F1mut-V10 vs 100% protection with T4 displayed F1mut-V upon re-challenge with 20,000 LD_50_ (Figure 9C)]. Thus, our data did not show consistent enhancement of proinflammatory cytokines by the V10 mutation, hence it is questionable that replacing native V with V10 mutant would lead to a significant beneficial effect in a new plague vaccine design. On the other hand, from a structural standpoint, deletion of the aa residues 271–300 disrupts the coiled coil bridge between the NH_2_- and COOH-domains of V [12], which would likely make V10 mutant a conformationally more flexible molecule and could adversely affect vaccine stability and efficacy.

Although humoral immune responses are critical for protection against plague, several studies have shown that cell-mediated immunity also plays important roles [23], [53], [54]. Wang et al. [53] established the role of CD8^+^ T cells in protection of mice against pneumonic plague evoked by *Y. pestis* KIM 1001 strain. This study corroborated the earlier report of Parent et al. [23], which concluded that plague vaccines that generate both humoral- and cell-mediated immune responses will be most effective. Likewise, Philipovskiy and Smiley (3) reported that mice vaccinated with a live *Y. pestis* vaccine primed both CD4+ and CD8^+^ T cells, which when passively transferred to naïve mice, provided protection against pulmonary *Y. pestis* infection [54]. The adjuvant-free T4 nanoparticle decorated F1mut-V induced robust F1- and V-specific antibody responses, as well as provided 100% protection to mice and rats against very high doses of *Y. pestis* challenge (Figures 7, 9 and 11). In addition, T4 delivery induced balanced T_H_1 and T_H_2 responses with a potent T_H_1 response, as evident from the induction of subclass IgG2a specific antibodies. Similar patterns were observed in our previous studies with the T4 displayed HIV-1 p24 immunogen [32]. Presumably, the large size of the T4 phage particle (capsid, 120 nm×86 nm; tail, 100 nm) allows for its efficient uptake by the antigen presenting cells and cross-presentation to both MHC-I and MHC-II molecules, stimulating both the humoral and cellular arms of the immune system. It is also possible that the T4 phage DNA containing CpG might potentially serve as a T_H_1-type of adjuvant. Indeed, studies have shown that F1-V vaccine adjuvanted with CpG or poly IC (also a T_H_1 type adjuvant), given by the intranasal route, induced both T_H_1 and T_H_2 responses, providing better protection to mice against bubonic and pneumonic plague [55], [56]. Thus, T4 might be a particularly useful platform for plague vaccine design since clearance of the *Y. pestis* bacterium may require a balanced response that is generally not seen with the current F1-V vaccines [46]. We also note that, although the mechanistic basis for T4 responses is currently unknown, no adverse effects to T4 vaccination have been observed in many preclinical studies performed in mouse, rat, rabbit, and rhesus macaque models [31], [57], [58], or in a human trial where T4 phage was given orally [59].

There has been a considerable urgency to develop a recombinant plague vaccine, but several concerns precluded licensing of current formulations. Our studies have established that the F1mut-V recombinant vaccine is efficacious and easily manufacturable and should be seriously considered as a next generation plague vaccine. Future studies would include preclinical evaluation of protection against *Y. pestis* infection in cynomolgus macaques as well as African Green monkeys, potentially leading to human clinical trials. Although the soluble F1mut-V vaccine adjuvanted with alum would be relatively easy to manufacture, the phage T4 nanoparticle-decorated F1mut-V vaccine offers certain advantages. First, the T4 formulation provided enhanced vaccine potency in small animal models. Second, the T4 vaccine would not require an extraneous adjuvant, and third, additional antigens from other biodefense pathogens, such as the protective antigen (PA) from *Bacillus anthracis* could be incorporated into the same formulation generating a dual vaccine against both inhalation anthrax and pneumonic plague. Our recent study demonstrated that the T4 displayed PA provided complete protection to Rhesus macaques against aerosol challenge with Ames spores of *B. anthracis* [51]. Fourth, the large interior of T4 head which has the capacity to package ∼171 kb DNA can also be used to deliver DNA vaccines [60]. By combining protein display outside and DNA packaging inside the T4 nanoparticles can simultaneously deliver vaccine antigen(s) as well as vaccine DNAs, similar to that of the prime-boost strategy, potentially inducing robust and long-lasting immune responses. Finally, such prime-boost vaccines could be targeted to antigen-presenting dendritic cells (DCs) by displaying a DC-specific ligand on the capsid using Hoc, further stimulating the cell-mediated immunity. One or two doses of such potent nanoparticle vaccines might be sufficient to afford protection against multiple biothreat agents. With the recent data demonstrating the proof of concept [60], we are currently developing these novel vaccine platforms, not only to defend against biowarfare pathogens but also to generate efficacious vaccines against complex infectious agents such as HIV-1 and malaria.

## Materials and Methods

### Ethics statement

This study was conducted in accordance with the recommendations in the Guide for the Care and Use of Laboratory Animals of the National Institutes of Health. The protocols were reviewed and approved by the Institutional Animal Care and Use Committees of the University of Texas Medical Branch, Galveston, TX, (Office of Laboratory Animal Welfare assurance number: A3314-01) and The Catholic University of America (Office of Laboratory Animal Welfare assurance number: A4431-01).

### DNA, bacteria, and bacteriophage

The T7 promoter containing *E. coli* expression vector pET28b (Novagen, MA) was used for recombinant plasmid construction. The template DNAs containing *Y. pestis* F1, V, or YscF were kindly provided by Dr. Richard Borschel from the Walter Reed Army Institute of Research (Silver Spring, MD). *E. coli* XL-10 Gold cells (Stratagene, CA) were used for the initial transformation of clones. The plasmid DNAs were then re-transformed into *E. coli* BL21 (DE3) RIPL (Novagen, MA) for expression of recombinant proteins. The Hoc^−^ Soc^−^ phage T4 was propagated on *E. coli* P301 and purified by CsCl gradient centrifugation.

### Construction of plague recombinant plasmids

The DNA encoding F1, V, or YscF were amplified by PCR using primers containing appropriate restriction site(s) (NheI/XhoI for F1 and YscF, and NheI/HindIII for V). The PCR products were purified, digested with appropriate restriction enzymes, and ligated with pET-28b vector DNA digested with the same restriction enzymes. The resulting plasmids had F1, V, or YscF coding sequences fused in-frame with the 23 aa vector sequence containing a hexa-histidine tag at the NH_2_-terminus. The YscF mutant, YscF35/67, which contained point mutations at aa 35 (Asn to Ser) and 67 (Ile to Thr) was amplified by overlap PCR [61] followed by digestion with NheI and XhoI enzymes. YscF35/67 DNA was then ligated into the linearized pET28b vector. The F1mut1, in which the first 14 aa residues were deleted and fused to the COOH-terminus with a two aa (SA) linker, was constructed by two rounds of PCR. The first round of PCR was performed to amplify F1 fragment in which the NH_2_-terminal 14 aa residues were deleted. This PCR product was used as a template for the second round of PCR using a forward primer containing NheI restriction site and a reverse primer containing the NH_2_-terminal 14 aa residues and XhoI restriction site. The PCR fragment was then inserted into NheI and XhoI linearized pET28b vector.

To construct F1mut2 in which aa residues 15 to 21 were duplicated at the COOH-terminus, a reverse primer with a 5′-tag corresponding to the 15 to 21 aa sequence and XhoI restriction site was used for PCR amplification. The F1mut2 fragment was then inserted into NheI and XhoI linearized pET28b vector. To construct F1-V recombinants, V was first amplified and inserted into BamHI and HindIII linearized pET28b vector to generate the pET-V clone. F1 and F1mut2 were amplified with primers containing NheI and BamHI restriction sites, digested with NheI and BamHI, and ligated with the pET-V vector DNA digested with the same restriction enzymes. The resulting F1-V and F1mut-V plasmids contained F1 or F1mut in-frame fusion with V and a 23-aa vector sequence containing the hexa-histidine sequence at the NH_2_-terminus of F1. The F1mut-V10 was amplified by overlap PCR using F1mut-V as the template and the mutated DNA was inserted into the NheI and HindIII linearized pET28b vector.

T4 Soc gene or RB69 Soc gene was fused with V, F1, or YscF with two aa (GS) linker by overlap PCR and the amplified DNA was inserted into the pET28b vector. The fused products V-T4 Soc, F1-T4 Soc, V-RB69Soc, and F1-RB69 Soc were further fused to YscF by overlap PCR to generate V-Soc (T4 or RB69)-YscF and F1-Soc (T4 or RB69)-YscF. Two aa residues, GS, were used as a linker between Soc and YscF. To construct F1-V-Soc clones, RB69 Soc gene was first amplified with end primers containing HindIII and XhoI restriction sites and inserted into the HindIII and XhoI linearized pET28b vector. This clone was then linearized by digestion with NheI and HindIII restriction enzymes. F1mut-V and F1mut-V10 DNAs were amplified by using the end primers containing NheI and HindIII restriction sites and inserted into the above plasmid. The resulting clones contained F1mut-V or F1mut-V10 fused in-frame to the NH_2_-terminus of RB69 Soc and also the flanking vector sequences containing two hexa-histidine tags at both NH_2_- and COOH-termini. The F1mut-V-Soc was then fused with YscF by overlap PCR with a two aa linker, GS, between Soc and YscF. All of the clones were sequenced (Retrogen, CA) and only the clones containing 100% sequence accuracy were used for protein purification. The primer sequences used and clones generated in these studies will be available upon request.

### Bioinformatics analysis

The structural models of F1, V, YscF, and T4 phage nanoparticle (Figure 1) were constructed using Chimera version1.4.1 [62]. The T cell epitopes were predicted using MetaMHC, a new web server which integrates the outputs of leading predictors by several popular ensemble strategies [63]. This was shown to generate statistically significant results that were more reliable than the individual predictors [63]. For the CD4^+^ T cell epitope prediction, F1 protein sequence was screened against 14 human MHC-II alleles. Peptides identified as positive ones by at least one predictor method were considered as potential CD4^+^ T cell epitopes. For the CD8^+^ T cell epitope prediction, F1 was screened against 57 human MHC-I alleles. Peptides identified as positive by at least one ensemble predictor approaches were considered to be potential CD8^+^ T cell epitopes. Default values were used for both the T cell epitope predictions.

### Over-expression, solubility analysis and purification of recombinant plague immunogens

The *E. coli* BL21 (DE3) RIPL cells harboring various plague recombinant plasmids constructed as above were induced with 1 mM IPTG for 1 to 2 h at 30°C. The cells were harvested by centrifugation at 4,000 *g* for 15 min at 4°C and the pellets were resuspended in 50 mM Tris-HCl (pH 8.0). Solubility analysis was carried out using bacterial protein extraction reagent (B-PER) (Thermo Fisher Scientific Inc., Rockford, IL). The cells were lysed with B-PER and centrifuged at 12,000 *g* for 10 min. The soluble supernatant and insoluble pellet fractions were analyzed by SDS-polyacrylamide gel electrophoresis (PAGE) as follows. The samples were boiled in a buffer containing SDS and β-mercaptoethanol, and were electrophoresed on a 12% or 15% (w/v) polyacrylamide gel. Since the protein aggregates will be dissociated into monomers under these conditions. The molecular weight differences observed in Figure 2B reflect sizes of the polypeptide chains of F1, F1mut1, and F1mut2. For example, F1mut1 and Fmut2 are approximately 1.6 kDa and 2.2 kDa larger than F1 because F1mut1 has a two amino acid linker (SA) and an eight amino acid His-tag (LEHHHHHH) (orange) at the C-terminus. F1mut2, in addition, has the duplicated T cell epitope (EPARITL) (blue) (Figure 2A).

For protein purification, the cells were resuspended in binding buffer (50 mM Tris-HCl pH 8.0, 300 mM NaCl and 20 mM imidazole) containing proteinase inhibitor cocktail (Roche, USA). The cells were lysed by French press (Aminco, IL, USA) at 12,000 psi and the soluble fractions containing the His-tagged fusion proteins were isolated by centrifugation at 34,000 *g* for 20 min. The supernatants were filtered through 0.22 µm filters (Sartorius Stedim Biotech, Germany) and loaded onto 1 ml HisTrap column (AKTA-prime, GE Healthcare) pre-equilibrated with 20 ml of binding buffer. After washing with the binding buffer containing 50 mM imidazole, the proteins were eluted with 20–500 mM linear imidazole gradient. The peak fractions containing the desired protein were concentrated by Amicon Ultra-4 centrifugal filtration (10 kDa cut-off; Millipore). The proteins were further purified by gel filtration on Hi-load 16/60 Superdex 200 column (AKTA-FPLC, GE Healthcare) in a buffer containing 20 mM Tris-HCl, pH 8.0 and 100 mM NaCl. The peak fractions containing the purified proteins were concentrated and stored at −80°C. The native F1 recombinant proteins were purified from the pellet containing the insoluble inclusion bodies. The pellet was dissolved in the binding buffer containing 8 M urea and loaded onto 1 ml HisTrap column (AKTA-prime, GE Healthcare) pre-equilibrated with the same buffer. The proteins were renatured by washing the column with a decreasing urea gradient (8 to 0 M) in the binding buffer. The bound proteins were then eluted with 20–500 mM linear imidazole gradient. If necessary, the peak fractions from the HisTrap column were concentrated by Amicon Ultra-4 centrifugal filtration (10 kDa cut-off). The proteins were further purified by gel filtration on Hi-load 16/60 Superdex 200 column as described above.

The levels of lipopolysaccharide (LPS) contamination in the purified recombinant *Y. pestis* antigens from *E. coli*; F1, LcrV, YscF, and F1mut-V, were determined using Endosafe PTS system (Charles River Laboratories International, Inc.,Wilmington, MA). This system consists of a handheld spectrophotometer and utilizes FDA approved disposable cartridges. At least three batches of each antigen were tested. The endotoxin levels ranged from 0.05 to 0.8 EU/ml, substantially lower than the maximum recommended in gene vectors and subunit vaccines, 10 and 20 EU/ml respectively, for preclinical research [64].

### *In vitro* binding of plague antigens on phage T4 capsid

*In vitro* binding of plague-Soc fusion protein on Hoc^−^ Soc^−^ T4 phage was carried out as previously described [29], [30], [32]. About 3×10^10^ phage particles were sedimented for 45 min at 34,000 *g* in LoBind Eppendorf tubes and resuspended in phosphate-buffered saline (PBS) buffer (pH 7.4). Various Soc fusion proteins were incubated with the resuspended Hoc^−^ Soc^−^ phage at 4°C for 45 min. The phage particles were sedimented at 34,000 *g* for 45 min and the supernatant containing the unbound protein was discarded. The phage pellet containing the bound plague antigen(s) was washed twice with excess buffer containing 20 mM Tris-HCl pH 8 and 100 mM NaCl. The final pellets were resuspended in PBS buffer (pH 7.4) and analyzed by SDS-PAGE. The gels were stained with Coomassie Blue R250 (Bio-Rad, USA) and the protein bands were quantified by laser densitometry (PDSI, GE Healthcare). The density of Soc fusion protein, gp23*, and gp18 (major tail sheath protein; 70 kDa) bands were determined for each lane separately and the copy number of bound plague antigen molecules per capsid was calculated using the known copy numbers of gp23* (930 molecules per capsid) or gp18 (138 molecules per capsid). A saturation binding curve relating the number of bound plague protein-Soc molecules per capsid (Y) and the concentration of unbound protein in the binding reaction (X) was generated by SigmaPlot software. The apparent K_d_ (association constant) and B_max_ (maximum copies of Soc fusion protein bound per capsid) were determined using the equation Y = B_max_X/(Kd+X) as programmed in the SigmaPlot software.

### Mouse immunizations and challenge

Six to eight weeks female Balb/c mice (17–20 g) were purchased from Jackson Laboratories (Bar Harbor, Maine) and randomly grouped and acclimated for 7 days. Equivalent amounts of plague immunogen molecules, either soluble or phage-bound, were used for each immunization. For immunization of soluble antigens, the purified proteins (10 µg/mouse/immunization) were adsorbed on alhydrogel (Brenntag Biosector, Denmark) containing 0.19 mg of aluminum per dose. For the T4 displayed antigens, the phage particles were directly used without any adjuvant (10 µg of plague antigen/mouse/immunization). On days 0, 21 and 42, mice were vaccinated via the intramuscular route. Alternate legs were used for each immunization. Blood was drawn from each animal on days 0 (pre-bleeds), 35 and 49 and the sera obtained were stored frozen at −70°C. On day 56, mice were intranasally challenged with *Y. pestis* CO92 BEI strain [65] using the indicated LD_50_. Animals were monitored and recorded twice daily for mortality or other symptoms for 48 to 88 days. The animals that survived were re-challenged intranasally at 48 or 88 days post-first challenge with the indicated LD_50_ and monitored twice daily for a further 48 days.

### Rat immunization and challenge

Female Brown Norway rats (50–75 g) were purchased from Charles River (Houston, TX). Upon arrival, animals were weighed and randomized into the treatment groups and were acclimated for several days before manipulation. The plague immunogens were prepared as described above. On days 0, 21 and 42, rats were vaccinated via the intramuscular route with 15 µg antigen in 50 µl PBS buffer. Alternate legs were used for each immunization. On day 56, animals were intranasally challenged with 5,000 LD_50_ of *Y. pestis* CO92 BEI strain and were monitored twice daily for 30 days and clinical symptoms of disease and survival recorded.

### Determination of IgG and IgG subtype antibodies

The IgG titers were determined by ELISA. Briefly, 96-well microtiter plates (Evergreen Scientific, Los Angeles, CA) were coated with 10 ng/well of purified F1, V, YscF, F1-V or F1mutV antigen at 4°C overnight. Following blocking and washing, sera from naïve and immunized mice were serially diluted and incubated with the affixed antigens for 1 h at room temperature. Following several washes, horseradish peroxidase (HRP)-conjugated goat anti-mouse IgG secondary antibody was added to the wells at a dilution of 1∶10,000. After incubation for 1 h at room temperature, the unbound antibody was removed and the wells were washed several times and the TMB (3,3′,5,5′-tetramethylbenzidine) substrate was added. Following a 20 min incubation to develop the color, the reaction was quenched by the addition of 2 N H_2_SO_4_ and the absorbance was read at 450 nm using an ELISA reader. For IgG subtypes, horseradish peroxidase-conjugated goat anti-mouse IgG1 or IgG2a secondary antibodies were used.

### Cytokines analysis

Seven days after the second boost (day 49), mice were sacrificed and spleens were harvested to prepare splenocytes using the lymphocyte separation medium. The isolated lymphocytes were adjusted to ∼5×10^6^ cells/ml and 1 ml of lymphocytes seeded into each well. Triplicate cultures from each group were stimulated with purified F1-V (10 µg/ml). Additional control stimulators included medium only and concanavalin A (5 µg/ml). After approximately 48 h incubation at 37°C in a humidified (5% CO_2_ in air) incubator, culture supernatants were collected. Cytokines were measured using a multiplex assay (Millipore, Billerica, MA). The results were analyzed in Prism and the statistical significance was determined by one way ANOVA with Bonferroni correction.

### Accession numbers of genes

F1 capsule antigen (*caf1*) [GeneID: 1172839, Sequence: NC_003134.1 (85950..86462)], *lcrV* [GeneID: 1172676; Sequence: NC_003131.1 (21935..22915, complement)], *yscF* [GeneID: 1172700, Sequence: NC_003131.1 (41026..41289)], and *soc* (RB69Soc) [GeneID: 1494143, Sequence: NC_004928.1 (14980..15216, complement)].

## Supporting Information

Text S1 Table S1. The predicted CD8^+^ T cell epitopes. CD8^+^ T cell epitopes were predicted using MetaMHC (http://www.biokdd.fudan.edu.cn/Service/MetaMHC.html) with default values. Peptides identified as positive by at least one ensemble predictor approach were considered to be potential CD8^+^ T cell epitopes. Highlighted cells indicate high ranking scores that predict a potential CD8^+^ T cell epitope. Table S2. The predicted CD4^+^ T cell epitopes. CD4^+^ T cell epitopes were predicted using MetaMHC (http://www.biokdd.fudan.edu.cn/Service/MetaMHC.html) with default values. Peptides identified as positive by at least one ensemble predictor approach were considered to be potential CD4^+^ T cell epitopes.
(DOC)
