# Supplementary material for: Mutated and Bacteriophage T4 Nanoparticle Arrayed F1-V Immunogens from Yersinia pestis as Next Generation Plague Vaccines
Source: PLoS Pathog. 2013 Jul 11;9(7):e1003495. doi: 10.1371/journal.ppat.1003495 (PMC3708895; doi:10.1371/journal.ppat.1003495)
Supplement: Text S1 — Table S1. The predicted CD8+ T cell epitopes. CD8+ T cell epitopes were predicted using MetaMHC (http://www.biokdd.fudan.edu.cn/Service/MetaMHC.html) with default values. Peptides identified as positive by at least one ensemble predictor approach were considered to be potential CD8+ T cell epitopes. Highlighted cells indicate high ranking scores that predict a potential CD8+ T cell epitope. Table S2. The predicted CD4+ T cell epitopes. CD4+ T cell epitopes were predicted using MetaMHC (http://www.biokdd.fudan.edu.cn/Service/MetaMHC.html) with default values. Peptides identified as positive by at least one ensemble predictor approach were considered to be potential CD4+ T cell epitopes. (DOC) [file ppat.1003495.s001.doc]

**Supporting Information**

**Mutated and bacteriophage T4 nanoparticle arrayed F1-V immunogens from *Yersenia pestis* as next generation plague vaccines**

Pan Tao^1^, Marthandan Mahalingham^1^, Michelle L. Kirtley^2^, Christina J. van Lier^2^, Jian Sha^2,4,5^, Linsey A. Yeager^2,5^, Ashok K. Chopra^2-5*^, and Venigalla B. Rao^1*^

^1^Department of Biology, The Catholic University of America, 620 Michigan Avenue NE, Washington, DC 20064, USA.

^2^Department of Microbiology and Immunology, ^3^Sealy Center for Vaccine Development, ^4^Institute of Human Infections and Immunity, and ^5^Galveston National Laboratory, The University of Texas Medical Branch, 301 University Blvd, Galveston, TX 77555, USA.

**Supporting Information contains two tables**

**Table S1. The predicted CD8^+^ T cell epitopes**

**Table S2. The predicted CD4^+^ T cell epitopes**

**Table S1. The predicted CD8^+^ T cell epitopes**

| Allele | Position | peptide | Methods used for epitope prediction | | | | | | |
| --- | --- | --- | --- | --- | --- | --- | --- | --- | --- |
|  |  |  | ANN | SMM | NetMHC | NetMHCPan | Consensus | PM | AvgTanh |
| HLA A*0101 | 138-146 | YTDAVTVTV | 59.3 | 138 | 117 | 94.4 | 0.999 | ∞ | 0.8421 |
|  | 62-70 | FTDAAGDPM | 325.9 | 539.9 | 1602 | 137.2 | 0.996 | 61.987 | 0.8149 |
|  | 6-14 | STTATATLV | 9916.2 | 2678.2 | 6400 | 2134.6 | 0.979 | 9.4645 | 0.7374 |
|  | 3-11 | LTASTTATA | 9123.5 | 2350.9 | 14883 | 2462.3 | 0.9775 | 8.2556 | 0.736 |
|  | 115-123 | ATGSQDFFV | 10097.4 | 3794.7 | 10875 | 2877.3 | 0.974 | 7.0794 | 0.7257 |
|  | 74-82 | FTSQDGNNH | 11247.4 | 8291.7 | 7860 | 4523.7 | 0.9738 | 4.4891 | 0.7047 |
|  | 15-23 | EPARITLTY | 15146 | 4479.6 | 19506 | 2814.7 | 0.9571 | 5.3529 | 0.687 |
|  | 55-63 | TTSTSVNFT | 11617. | 6486.9 | 5267 | 13117 | 0.9567 | 1.5686 | 0.6576 |
| HLA A*0201 | 138-146 | YTDAVTVTV | 15 | 67.4 | 15 | 10.9 | 0.9982 | 33.9982 | 0.7844 |
|  | 115-123 | ATGSQDFFV | 552 | 526.2 | 196 | 350.5 | 0.9726 | 10.7835 | 0.696 |
| HLA A*0202 | 138-146 | YTDAVTVTV | 52.7 | 111.8 | 70 | 19.1 | 0.984 | 17.2995 | 0.7035 |
|  | 115-123 | ATGSQDFFV | 140.4 | 218.1 | 103 | 328.2 | 0.9691 | 9.4752 | 0.663 |
|  | 106-114 | NLVGDDVVL | 231.2 | 128.6 | 216 | 1028.5 | 0.9694 | 6.7764 | 0.6441 |
| HLA A*0203 | 3-11 | LTASTTATA | 64 | 283.8 | 39 | 66.4 | 0.9796 | 14.9505 | 0.6862 |
|  | 138-146 | YTDAVTVTV | 185.1 | 280.1 | 54 | 40.6 | 0.9729 | 13.3352 | 0.6779 |
|  | 9-17 | ATATLVEPA | 153.1 | 279.9 | 88 | 282.7 | 0.9666 | 9.7571 | 0.6611 |
| HLA A*0204 | 138-146 | YTDAVTVTV |  |  | 84 | 32.8 | 0.9965 | � | 0.84 |
|  | 115-123 | ATGSQDFFV |  |  | 64 | 329.5 | 0.9924 | � | 0.7991 |
|  | 6-14 | STTATATLV |  |  | 316 | 1289.2 | 0.9734 | � | 0.7298 |
| HLA A*0206 | 138-146 | YTDAVTVTV | 8 | 21.7 | 11 | 6.8 | 0.9972 | 30.8606 | 0.7174 |
|  | 115-123 | ATGSQDFFV | 111.2 | 169.1 | 92 | 108.1 | 0.9767 | 13.0157 | 0.6561 |
|  | 6-14 | STTATATLV | 105.4 | 197.8 | 109 | 196.2 | 0.974 | 11.7425 | 0.6501 |
|  | 62-70 | FTDAAGDPM | 105.3 | 155 | 188 | 202 | 0.9704 | 11.1491 | 0.6483 |
| HLA  A*0211 | 138-146 | YTDAVTVTV | 3 | 12 | 3 | 2.2 | 0.9948 | � | 0.7157 |
| HLA A*0212 | 138-146 | YTDAVTVTV | 7.5 | 58.9 | 4 | 6.4 | 0.9957 | � | 0.7545 |
|  | 40-48 | ELLVGTLTL | 101.8 | 730 | 16 | 779.8 | 0.9667 | � | 0.6778 |
|  | 115-123 | ATGSQDFFV | 226.3 | 488.7 | 442 | 156.2 | 0.9656 | � | 0.6732 |
|  | 106-114 | NLVGDDVVL | 247.9 | 39.7 | 42 | 1517.4 | 0.9749 | � | 0.6711 |
| HLA A*0216 | 138-146 | YTDAVTVTV | 7.9 | 11.2 | 8 | 4.4 | 0.9975 | � | 0.7406 |
|  | 115-123 | ATGSQDFFV | 21.3 | 106.5 | 14 | 47.8 | 0.982 | � | 0.7051 |
|  | 40-48 | ELLVGTLTL | 50.5 | 183 | 6 | 419.3 | 0.9769 | � | 0.68 |
| HLA A*0219 | 138-146 | YTDAVTVTV | 24.8 | 144.3 | 6 | 6.4 | 0.9961 | � | 0.786 |
|  | 40-48 | ELLVGTLTL | 14.5 | 73.9 | 20 | 948.5 | 0.9924 | � | 0.7409 |
|  | 106-114 | NLVGDDVVL | 1750.7 | 69.4 | 437 | 2545.1 | 0.9702 | � | 0.6701 |
| HLA  A*0250 | 138-146 | YTDAVTVTV | 9.4 | 62.6 |  | 93 | 0.9824 | � | 0.6073 |
| HLA A*0301 | 44-52 | GTLTLGGYK | 126.9 | 257.8 | 64 | 110.4 | 0.997 | 23.4818 | 0.7992 |
|  | 124-132 | RSIGSKGGK | 214.8 | 262 | 216 | 478.9 | 0.9941 | 16.634 | 0.7735 |
|  | 129-137 | KGGKLAAGK | 2747.1 | 1541 | 3933 | 2306.2 | 0.9718 | 3.4371 | 0.6822 |
|  | 121-129 | FFVRSIGSK | 7891 | 2239.3 | 1377 | 2222.7 | 0.9674 | 2.4575 | 0.6512 |
| HLA A*2402 | 66-74 | AGDPMYLTF | 4777.4 | 1719.5 | 17696 | 6562.3 | 0.9758 | -0.0957 | 0.6669 |
|  | 113-121 | VLATGSQDF | 4354.9 | 2130.2 | 9448 | 23998.5 | 0.9675 | -0.6753 | 0.63 |
| HLA A*2403 | 70-78 | MYLTFTSQD | 429.7 | 14.1 | 5500 | 4652.3 | 0.9702 | ∞ | 0.6988 |
|  | 66-74 | AGDPMYLTF | 4908.1 | 657.3 | 6978 | 562.4 | 0.9502 | ∞ | 0.6667 |
|  | 76-84 | SQDGNNHQF | 6312.4 | 1996.8 | 8479 | 280.8 | 0.9417 | ∞ | 0.6609 |
| HLA A*2501 | 38-46 | DTELLVGTL | 2226.2 | 144.8 |  | 5822 | 0.9912 | � | 0.712 |
|  | 15-23 | EPARITLTY | 2130.9 | 983.7 |  | 489.2 | 0.9916 | � | 0.7099 |
|  | 111-119 | DVVLATGSQ | 1974.1 | 838 |  | 15236.1 | 0.9866 | � | 0.6961 |
| HLA A*2601 | 15-23 | EPARITLTY | 2018.5 | 998.8 | 3308 | 181.3 | 0.9869 | 49.4191 | 0.7747 |
|  | 111-119 | DVVLATGSQ | 4874.1 | 1326.4 | 14407 | 3963.9 | 0.9785 | 14.8468 | 0.6999 |
|  | 62-70 | FTDAAGDPM | 13964.4 | 1384.7 | 15541 | 1849.9 | 0.9625 | 10.934 | 0.6612 |
| HLA A*2602 | 15-23 | EPARITLTY | 497.2 | 45.4 | 341 | 28.8 | 0.9863 | � | 0.7377 |
|  | 62-70 | FTDAAGDPM | 9574.8 | 98.6 | 255 | 123.4 | 0.9826 | � | 0.6744 |
|  | 38-46 | DTELLVGTL | 3879.3 | 144.9 | 727 | 448.9 | 0.9746 | � | 0.6701 |
|  | 111-119 | DVVLATGSQ | 753 | 496.8 | 23192 | 686.8 | 0.954 | � | 0.6666 |
| HLA A*2603 | 40-48 | ELLVGTLTL | 709.4 | 1251.8 |  | 22809.6 | 0.9576 | � | 0.6582 |
|  | 62-70 | FTDAAGDPM | 3008.3 | 208.3 |  | 3780.2 | 0.9818 | � | 0.6514 |
|  | 15-23 | EPARITLTY | 4313.2 | 308.5 |  | 1759.7 | 0.9874 | � | 0.6448 |
|  | 111-119 | DVVLATGSQ | 3972 | 3400.1 |  | 7218.6 | 0.97 | � | 0.6188 |
| HLA A*2902 | 15-23 | EPARITLTY | 817.4 | 225.2 | 819 | 330.8 | 0.9846 | 7.8134 | 0.7289 |
|  | 63-71 | TDAAGDPMY | 294.1 | 289.8 | 10993 | 6113.4 | 0.9609 | 1.925 | 0.6728 |
| HLA A*3001 | 16-24 | PARITLTYK | 13.4 | 50.5 | 241 | 44.7 | 0.9877 | 79.8723 | 0.7132 |
|  | 124-132 | RSIGSKGGK | 80 | 124.6 | 40 | 25.5 | 0.9839 | ∞ | 0.7067 |
|  | 44-52 | GTLTLGGYK | 113.6 | 111.5 | 447 | 79.9 | 0.9746 | 53.7159 | 0.6804 |
|  | 52-60 | KTGTTSTSV | 343.5 | 246.5 | 4727 | 78.7 | 0.9538 | 43.5615 | 0.651 |
| HLA A*3002 | 43-51 | VGTLTLGGY | 83.5 | 123.7 | 11113 | 766.9 | 0.9816 | 8.8231 | 0.7082 |
|  | 130-138 | GGKLAAGKY | 340.2 | 120.4 | 17505 | 1539.2 | 0.97 | 4.012 | 0.6751 |
|  | 44-52 | GTLTLGGYK | 412.6 | 544 | 201 | 244.9 | 0.9704 | 17.395 | 0.6682 |
| HLA A*3201 | 52-60 | KTGTTSTSV | 126 | 159.6 |  | 226.8 | 0.986 | 4.5679 | 0.7168 |
|  | 54-62 | GTTSTSVNF | 191.7 | 189 |  | 280.8 | 0.9842 | 3.8872 | 0.7078 |
|  | 138-146 | YTDAVTVTV | 127.1 | 657.1 |  | 211.7 | 0.9868 | 3.9987 | 0.7051 |
|  | 118-126 | SQDFFVRSI | 229.3 | 152.9 |  | 357.6 | 0.9804 | 3.6174 | 0.7051 |
|  | 11-19 | ATLVEPARI | 530.4 | 229.1 |  | 1193.7 | 0.971 | 1.7813 | 0.6793 |
|  | 40-48 | ELLVGTLTL | 277.7 | 468.9 |  | 3716.4 | 0.948 | 1.2904 | 0.6759 |
|  | 21-29 | LTYKEGAPI | 1060 | 1051.9 |  | 105.1 | 0.9694 | 2.4168 | 0.6706 |
|  | 5-13 | ASTTATATL | 420.5 | 663.4 |  | 2591.9 | 0.9314 | 1.0012 | 0.6686 |
| HLA A*3301 | 10-18 | TATLVEPAR | 203.2 | 276.6 | 145 | 491 | 0.9861 | 24.3677 | 0.7268 |
| HLA A*6801 | 10-18 | TATLVEPAR | 12.3 | 21.3 | 5 | 45.3 | 0.9916 | 26.7757 | 0.7406 |
|  | 116-124 | TGSQDFFVR | 277.4 | 112.5 | 143 | 345.2 | 0.9682 | 8.9889 | 0.6706 |
| HLA A*6802 | 138-146 | YTDAVTVTV | 11.3 | 45.8 | 33 | 14.6 | 0.9977 | 35.8551 | 0.7478 |
|  | 6-14 | STTATATLV | 21 | 74.6 | 48 | 16.7 | 0.9952 | 31.6877 | 0.7379 |
|  | 3-11 | LTASTTATA | 57.3 | 199.7 | 143 | 30.5 | 0.9869 | 23.3999 | 0.714 |
|  | 9-17 | ATATLVEPA | 13.6 | 158.7 | 46 | 254.6 | 0.9898 | 25.4081 | 0.7134 |
|  | 64-72 | DAAGDPMYL | 53.9 | 176.6 | 50 | 152.9 | 0.9878 | 21.2437 | 0.7059 |
|  | 58-66 | TSVNFTDAA | 79.1 | 275.1 | 57 | 108.6 | 0.9864 | 20.2662 | 0.7026 |
|  | 55-63 | TTSTSVNFT | 71.3 | 370.1 | 180 | 301.2 | 0.9786 | 16.061 | 0.6862 |
|  | 115-123 | ATGSQDFFV | 245 | 364.1 | 186 | 242.7 | 0.9777 | 13.1418 | 0.6747 |
| HLA A*6901 | 138-146 | YTDAVTVTV | 5.1 | 17 | 6 | 6.1 | 0.9996 | ∞ | 0.7974 |
|  | 6-14 | STTATATLV | 37 | 99.8 | 85 | 31.4 | 0.9954 | ∞ | 0.7522 |
|  | 62-70 | FTDAAGDPM | 62.3 | 164.3 | 133 | 173.2 | 0.9902 | ∞ | 0.7268 |
|  | 3-11 | LTASTTATA | 253.2 | 406.9 | 93 | 72.5 | 0.988 | ∞ | 0.7166 |
|  | 40-48 | ELLVGTLTL | 48.2 | 312.8 | 60 | 1953.4 | 0.9872 | ∞ | 0.6987 |
|  | 21-29 | LTYKEGAPI | 219.7 | 425.8 | 196 | 441.6 | 0.9811 | ∞ | 0.6951 |
|  | 64-72 | DAAGDPMYL | 240.4 | 326.5 | 107 | 760.4 | 0.9808 | ∞ | 0.6914 |
|  | 115-123 | ATGSQDFFV | 913 | 233.4 | 392 | 161.5 | 0.9822 | ∞ | 0.6856 |
| HLA A*8001 | 15-23 | EPARITLTY | 124.9 | 229.1 |  | 5377.4 | 0.9902 | � | 0.7414 |
|  | 43-51 | VGTLTLGGY | 4753 | 905 |  | 5409.9 | 0.9706 | � | 0.6655 |
| HLA B*0702 | 99-107 | SPKVNGENL | 228.5 | 212.6 | 67 | 171.5 | 0.9934 | 27.4616 | 0.7759 |
|  | 13-21 | LVEPARITL | 1215.9 | 992.8 | 719 | 1098.3 | 0.98 | 12.0184 | 0.7088 |
| HLA B*0801 | 40-48 | ELLVGTLTL | 2672.6 | 972.1 | 12249 | 2018.3 | 0.9656 | 12.651 | 0.6924 |
| HLA B*0802 | 99-107 | SPKVNGENL | 11002 | 7279 | 34987 | 32977.9 | 0.9864 | � | 0.7136 |
|  | 15-23 | EPARITLTY | 15131.9 | 37002.1 | 47882 | 28063.9 | 0.8898 | � | 0.6892 |
| HLA B*0803 | 99-107 | SPKVNGENL | 10361 | 1984.5 |  |  | 0.9912 | � | 0.7064 |
|  | 113-121 | VLATGSQDF | 15753.7 | 8917.4 |  |  | 0.9409 | � | 0.6339 |
| HLA B*1501 | 113-121 | VLATGSQDF | 136.5 | 461.7 | 186 | 75.5 | 0.9918 | 19.6771 | 0.7104 |
|  | 21-29 | LTYKEGAPI | 358.2 | 556.7 | 6195 | 560.1 | 0.9733 | 8.8593 | 0.6541 |
|  | 76-84 | SQDGNNHQF | 1347.4 | 636.9 | 392 | 1836.2 | 0.9626 | 6.8116 | 0.6345 |
| HLA B*1502 | 113-121 | VLATGSQDF | 64.2 | 45.1 |  | 426.4 | 0.9846 | � | 0.6889 |
|  | 40-48 | ELLVGTLTL | 1719.6 | 40.2 |  | 4329.9 | 0.9742 | � | 0.6078 |
| HLA B*1503 | 136-144 | GKYTDAVTV | 29.6 | 17.1 |  | 100.1 | 0.9726 | 6.5537 | 0.6379 |
|  | 113-121 | VLATGSQDF | 50.6 | 62.9 |  | 40.6 | 0.983 | 6.1082 | 0.6327 |
| HLA B*1509 | 81-89 | NHQFTTKVI | 975.7 | 314.6 |  | 3588 | 0.9972 | � | 0.7275 |
| HLA B*1517 | 5-13 | ASTTATATL | 8.2 | 2.4 |  | 11.7 | 0.9946 | � | 0.6826 |
|  | 21-29 | LTYKEGAPI | 14.6 | 2.4 |  | 6.3 | 0.9966 | � | 0.6791 |
|  | 54-62 | GTTSTSVNF | 6.7 | 20.6 |  | 7.5 | 0.996 | � | 0.6748 |
|  | 11-19 | ATLVEPARI | 18.9 | 9.2 |  | 113.8 | 0.9858 | � | 0.6589 |
| HLA B*1801 | 15-23 | EPARITLTY | 2718.7 | 3623.5 | 3568 | 967.2 | 0.9762 | -0.6173 | 0.6864 |
|  | 39-47 | TELLVGTLT | 4635.5 | 2931.7 | 4242 | 2985.1 | 0.9686 | -2.4987 | 0.651 |
| HLA B*2705 | 93-101 | SRDFDISPK | 1778.7 | 608.6 | 2340 | 1789.4 | 0.9728 | ∞ | 0.668 |
|  | 17-25 | ARITLTYKE | 2303.8 | 1806.8 | 2470 | 1058.6 | 0.9648 | ∞ | 0.6639 |
| HLA B*3501 | 15-23 | EPARITLTY | 7.7 | 6.2 | 7 | 3.8 | 0.9998 | 46.8204 | 0.7968 |
|  | 62-70 | FTDAAGDPM | 75.8 | 127.7 | 64 | 83.4 | 0.9919 | 22.3804 | 0.7274 |
|  | 68-76 | DPMYLTFTS | 131.1 | 126.4 | 244 | 286.1 | 0.9878 | 16.4819 | 0.701 |
|  | 114-122 | LATGSQDFF | 219.5 | 234.7 | 410 | 960.7 | 0.9804 | 12.0409 | 0.6738 |
|  | 63-71 | TDAAGDPMY | 919.1 | 417.9 | 330 | 5103 | 0.9728 | 6.4352 | 0.6286 |
| HLA B*3801 | 81-89 | NHQFTTKVI | 13519.1 | 1538.5 |  | 9323 | 0.986 | � | 0.6706 |
|  | 76-84 | SQDGNNHQF | 10354.3 | 31596.3 |  | 8218.1 | 0.9864 | � | 0.6638 |
|  | 23-31 | YKEGAPITI | 22462.2 | 5741.3 |  | 2616.9 | 0.9576 | � | 0.6413 |
|  | 13-21 | LVEPARITL | 12930.8 | 16142.5 |  | 21508.9 | 0.9318 | � | 0.6298 |
| HLA B*3901 | 23-31 | YKEGAPITI | 92.8 | 448.5 | 1006 | 130.3 | 0.994 | � | 0.7821 |
|  | 76-84 | SQDGNNHQF | 591.4 | 888.1 | 923 | 1546.4 | 0.9824 | � | 0.7411 |
|  | 81-89 | NHQFTTKVI | 829.9 | 514.5 | 3308 | 1208.6 | 0.9856 | � | 0.7363 |
|  | 106-114 | NLVGDDVVL | 498.2 | 150.2 | 10302 | 3234.2 | 0.9788 | � | 0.7294 |
|  | 40-48 | ELLVGTLTL | 657 | 859.5 | 3849 | 2156.6 | 0.9814 | � | 0.7276 |
|  | 138-146 | YTDAVTVTV | 535 | 2034.1 | 3891 | 1988.3 | 0.9764 | � | 0.7268 |
|  | 118-126 | SQDFFVRSI | 3188 | 2262.4 | 11730 | 482.6 | 0.9668 | � | 0.7021 |
|  | 136-144 | GKYTDAVTV | 1996.9 | 2257.1 | 27576 | 3282.4 | 0.9532 | � | 0.6833 |
|  | 82-90 | HQFTTKVIG | 1740.5 | 903 | 31399 | 5769.3 | 0.9484 | � | 0.6763 |
|  | 5-13 | ASTTATATL | 2971.7 | 6783.5 | 7131 | 5936.5 | 0.945 | � | 0.6582 |
| HLA B*4001 | 24-32 | KEGAPITIM | 633.3 | 324.1 | 1947 | 323 | 0.993 | 49.5406 | 0.7902 |
|  | 104-112 | GENLVGDDV | 324.2 | 212.4 | 549 | 1126 | 0.9942 | ∞ | 0.7883 |
|  | 76-84 | SQDGNNHQF | 7692.4 | 2253.1 | 3933 | 9227.5 | 0.9732 | 6.7596 | 0.6504 |
| HLA B*4002 | 24-32 | KEGAPITIM | 278.8 | 168.4 | 1968 | 257.6 | 0.9943 | 7.5012 | 0.797 |
|  | 94-102 | RDFDISPKV | 180.5 | 182.6 | 9146 | 114.8 | 0.9957 | 8.4816 | 0.7955 |
| HLA B*4402 | 104-112 | GENLVGDDV | 1786.1 | 1299.1 | 3766 | 5638.9 | 0.9873 | -0.5968 | 0.7061 |
|  | 24-32 | KEGAPITIM | 2228.9 | 4184.8 | 3344 | 4613.7 | 0.9866 | -1.1406 | 0.6988 |
|  | 39-47 | TELLVGTLT | 6837.2 | 4189.4 | 8297 | 3091.9 | 0.967 | -3.4574 | 0.6657 |
| HLA B*4403 | 104-112 | GENLVGDDV | 1315.4 | 1032.2 | 2055 | 2673.4 | 0.9873 | 0.9949 | 0.7412 |
|  | 39-47 | TELLVGTLT | 3615 | 3983.7 | 2033 | 1195.1 | 0.981 | 0.2632 | 0.7263 |
|  | 24-32 | KEGAPITIM | 3337.2 | 2480.6 | 3646 | 1339.2 | 0.9762 | -0.2196 | 0.7244 |
| HLA B*4501 | 104-112 | GENLVGDDV | 590.1 | 512.4 | 1183 | 864.6 | 0.9891 | 6.1365 | 0.7583 |
|  | 39-47 | TELLVGTLT | 1293 | 493.9 | 6263 | 524.2 | 0.9823 | 4.0199 | 0.7377 |
|  | 24-32 | KEGAPITIM | 3597 | 5363.6 | 6829 | 3068.3 | 0.9686 | -1.167 | 0.6769 |
|  | 1-9 | ADLTASTTA | 4385.1 | 665.2 | 6756 | 11184.4 | 0.967 | -1.7047 | 0.6456 |
| HLA B*4601 | 62-70 | FTDAAGDPM | 10726.5 | 1559.4 |  | 7426.4 | 0.9924 | � | 0.6837 |
|  | 134-142 | AAGKYTDAV | 11109.7 | 4486.5 |  | 23447.9 | 0.9854 | � | 0.6623 |
|  | 21-29 | LTYKEGAPI | 10059.8 | 40641 |  | 3883.3 | 0.9884 | � | 0.6606 |
|  | 138-146 | YTDAVTVTV | 14000.4 | 14907.6 |  | 12652.9 | 0.9708 | � | 0.6284 |
| HLA B*4801 | 82-90 | HQFTTKVIG | 5396.5 | 6738 |  | 25374.6 | 0.9632 | � | 0.648 |
| HLA B*5101 | 15-23 | EPARITLTY | 9933.7 | 9019.8 | 2722 | 4595.8 | 0.9884 | -3.8522 | 0.7363 |
|  | 21-29 | LTYKEGAPI | 13786.2 | 1897.6 | 11858 | 2786.3 | 0.9867 | -4.5475 | 0.7234 |
|  | 68-76 | DPMYLTFTS | 19139.2 | 12211.3 | 4676 | 13716.9 | 0.964 | -6.6504 | 0.6432 |
|  | 99-107 | SPKVNGENL | 16209.7 | 8779.6 | 10758 | 16892.2 | 0.9688 | -7.2877 | 0.6411 |
| HLA B*5301 | 15-23 | EPARITLTY | 64.4 | 132.5 | 16 | 11.6 | 0.9988 | 22.6049 | 0.8709 |
|  | 114-122 | LATGSQDFF | 1316.3 | 435.3 | 8208 | 3083 | 0.9822 | 0.3717 | 0.7594 |
|  | 68-76 | DPMYLTFTS | 6521.6 | 1499.6 | 6540 | 3350.5 | 0.9707 | -2.0248 | 0.7135 |
| HLA B*5401 | 68-76 | DPMYLTFTS | 1187.7 | 3591.9 | 1422 | 1200.2 | 0.9923 | 5.0207 | 0.7715 |
|  | 15-23 | EPARITLTY | 11348.1 | 4909.2 | 13947 | 1004.6 | 0.9534 | -1.6201 | 0.6938 |
|  | 133-141 | LAAGKYTDA | 4189.1 | 256 | 2969 | 18742.4 | 0.985 | 0.2011 | 0.6846 |
|  | 3-11 | LTASTTATA | 8053.3 | 208.7 | 15208 | 7990.1 | 0.9726 | -1.6094 | 0.6826 |
|  | 27-35 | APITIMDNG | 3514.5 | 941.3 | 2123 | 30694.5 | 0.9767 | -0.4824 | 0.6633 |
| HLA B*5701 | 114-122 | LATGSQDFF | 591 | 986.9 | 16054 | 2744.5 | 0.9814 | 9.3313 | 0.7484 |
|  | 21-29 | LTYKEGAPI | 3004.6 | 3996.2 | 27279 | 1461.1 | 0.9637 | 2.7728 | 0.7142 |
|  | 54-62 | GTTSTSVNF | 11032.9 | 3347.2 | 2551 | 746.9 | 0.9736 | 6.411 | 0.6923 |
| HLA B*5801 | 114-122 | LATGSQDFF | 34 | 248 | 759 | 490.4 | 0.9892 | ∞ | 0.7752 |
|  | 54-62 | GTTSTSVNF | 1243 | 692.7 | 1347 | 371.7 | 0.9816 | ∞ | 0.7332 |
|  | 5-13 | ASTTATATL | 1739 | 1663.3 | 14099 | 1009.3 | 0.9636 | ∞ | 0.6952 |
| HLA B*7301 | 1-9 | ADLTASTTA | 33384.6 | 237.6 |  | 30447.1 | 0.85 | � | 0.5741 |

CD8^+^ T cell epitopes were predicted using MetaMHC (<http://www.biokdd.fudan.edu.cn/Service/MetaMHC.html> ) with default values. Peptides identified as positive by at least one ensemble predictor approach were considered to be potential CD8^+^ T cell epitopes. Highlighted cells indicate high ranking scores that predict a potential CD8^+^ T cell epitope.

**Table S2. The predicted CD4^+^ T cell epitopes**

| **Position** | **Peptide** | **Allele** | **Positive prediction by the indicated approaches** |
| --- | --- | --- | --- |
| 101-121 | KVNGENLVGDDVVLATGSQDF | DRB1_0301 | TEPITOPE, SMM-align, Consensus, PM & AvgTanh |
| 83-101 | QFTTKVIGKDSRDFD ISPK | DRB1_0301 | SMM-align |
| 101-121 | KVNGENLVGDDVVLATGSQDF | DRB1_0401 | SMM-align, Consensus, and MetaSVMp |
| 68-86 | DPMYLTFTSQDGNNH QFTT | DRB1_0401 | LA Kernel, SMM-align, Consensus, PM & AvgTanh and MetaSVMp |
| 36-63 | NIDTELLVGTLTLGGYKTGTTSTSVNFT | DRB1_0401 | SMM-align, Consensus, PM & AvgTanh and MetaSVMp |
| 135-149 | AGKYTDAVTVTVSNQ | DRB1_0401 | LA Kernel |
| 36-54 | NIDTELLVGTLTLGGYKTG | DRB1_0404 | SMM-align, Consensus, PM & AvgTanh and MetaSVMp |
| 68-83 | DPMYLTFTSQDGNNHQ | DRB1_0404 | LA Kernel, Consensus and PM |
| 64-83 | DAAGDPMYLTFTSQDGNNHQ | DRB1_0405 | SMM-align, PM & AvgTanh and MetaSVMp |
| 1-18 | ADLTASTTATATLVE PAR | DRB1_0701 | LA Kernel, SMM-align, Consensus and PM |
| 133-149 | LAAGKYTDAVTVTVSNQ | DRB1_0701 | LA Kernel and Consensus |
| 47-63 | TLGGYKTGTTSTSVNFT | DRB1_0701 | SMM-align |
| 115-135 | ATGSQDFFVRSIGSKGGKLAA | DRB1_0802 | TEPITOPE |
| 17-35 | ARITLTYKEGAPITIMDNG | DRB1_0901 | SMM-align and MetaSVMp |
| 1-15 | ADLTASTTATATLVE | DRB1_0901 | Consensus |
| 34-48 | NGNIDTELLVGTLTLGGYKTGT | DRB1_1302 | LA Kernel and MetaSVMp |
|  | No positive results | DRB1_1101 |  |
| 39-54 | TELLVGTLTLGGYKTG | DRB1_1501 | SMM-align and MetaSVMp |
| 116-135 | TGSQDFFVRSIGSKGGKLAA | DRB1_1501 | SMM-align |
| 83-120 | QFTTKVIGKDSRDFDISPKVNGENLVGDDVVLATGSQD | DRB3_0101 | LA Kernel, SMM-align, Consensus, PM & AvgTanh and MetaSVMp |
| 33-47 | DNGNIDTELLVGTLT | DRB3_0101 | LA Kernel |
|  | No positive results | DRB4_0101 |  |
|  | No positive results | DRB5_0101 |  |

CD4^+^ T cell epitopes were predicted using MetaMHC (<http://www.biokdd.fudan.edu.cn/Service/MetaMHC.html> ) with default values. Peptides identified as positive by at least one ensemble predictor approach were considered to be potential CD4^+^ T cell epitopes.
